# Phosphatidylserine modulation influences syncytia formation in human breast cancer cell lines

**DOI:** 10.1186/s12885-026-17341-z

**Published:** 2026-11-10

**Authors:** Mareike Sieler, Leonie Huwa, Silvia Keil, Maja Kneissig, Thomas Dittmar

**Affiliations:** Immunology and Tumor Biology, Center for Biomedical Education and Research (ZBAF), Witten/Herdecke University, Stockumer Str. 10, 58453 Witten, Germany

**Keywords:** Cell fusion, Breast cancer, Syncytin-1, Dual split protein assay, Phosphatidylserine, Ano6/TMEM16F, Atp8a1, Niclosamide, Clofazimine, Salinomycin, Hexachlorophene

## Abstract

**Background:**

Cell-cell fusion is a biological process, crucial for the development and homeostasis of tissue. In contrast, cell fusion is also pathologically associated with viral infections or cancer cell fusion. While physiological cell fusion is strictly regulated, is it not clear how and why cancer cells fuse with other cells. Since the resulting cancer hybrid cells can have new malignant properties, like chemotherapeutic resistance or an enhanced metastatic potential, it is of interest to get to know how cancer cell fusion takes place.

**Methods:**

A breast cancer cell fusion model, relying on the dual split protein cell fusion assay and overexpression of the fusogen Syncytin-1, was used to study the impact of the membrane lipid phosphatidylserine (PS) and lipid distributing proteins. Therefore, the breast epithelial cell line M13SV1 was cocultured with the breast cancer cell lines MDA-MB-231 or HS578T. To mask PS, we added the PS-binding protein Annexin V in the coculturing medium. The most prominent expressed scramblase Ano6/TMEM16F was investigated using the clinically used substances Niclosamide, Clofazimine, Salinomycin and Hexachlorophene to indirectly or directly inhibit the protein’s activity. In addition, Ano6 was knocked-out using CRISPR/Cas9. The flippase Atp8a1 was simultaneously overexpressed with its cofactor CDC50a and assays assessing the tumorigenic properties as well as the cell fusion capacity of the differently treated or altered cells were done.

**Results:**

While Annexin V was able to significantly decrease Syncytin-1-mediated cell fusion, the four substances Niclosamide, Clofazimine, Salinomycin and Hexachlorophene did not affect the amount of phosphatidylserine on the cells’ surface or their capacity to fuse with other cells. The Ano6/TMEM16F KO as well as the Atp8a1-CDC50a overexpression showed rather non-significant effects on the cell fusion.

**Conclusion:**

Although the effects were mainly non-significant, it was shown, that Syncytin-1-mediated cell fusion could be decreased when M13SV1 cells overexpressing Syncytin-1 were overexpressing Atp8a1-CDC50a, suggesting that less PS in the membrane of these cells is causing the decrease in cell fusion. Vice versa, the loss of Ano6 or the overexpression of Atp8a1-CDC50a in the breast cancer cell lines was able to increase Syncytin-1-mediated cell fusion. This suggests the hypothesis that the localization of PS during fusion may be dynamic and that externalized PS may enhance the function of Syncytin-1 on the expressing cell, while it appears to disrupt cell fusion when exposed on the neighboring cell.

**Supplementary Information:**

The online version contains supplementary material available at https://doi.org/10.1186/s12885-026-17341-z.

## Background

Cell fusion is an essential process in biology, which is of great importance both for the creation of new life and for the homeostasis of organisms [1, 2]. The fusion of sperm and egg cells, the development of the placenta, and the development and maintenance of muscles and bones are among the most important processes in which cell fusion takes place [3–6]. In contrast, cell fusion also takes place in pathological conditions, like in viral infections or in cancer [7–10]. To date, many components of cell fusion are known, but the complete process has not yet been fully elucidated, especially for the fusion of cancer cells with other cells.

Different proteins and molecular players regulate cell fusion [8, 11, 12]. For example, it is known that fusogenic proteins can catalyze it and that their activity can be influenced by inflammatory processes or hypoxia [11, 12]. A frequently described pair of fusogen and receptor is the human endogenous retroviral (HERV) protein Syncytin-1 and alanine serine cysteine transporter 2 (ASCT2), which can bring neighboring cells in a close proximity for cell fusion [13]. While the expression of Syncytin-1 is strongly timely regulated [14, 15], meaning that it is only found on cells that are supposed to fuse, such as trophoblasts or myoblasts, the receptor and amino acid transporter ASCT2 is expressed in specific tissues and also highly expressed in tumor tissue [16, 17]. The expression of Syncytin-1 was also found in various tumors, leading to cancer cell fusion and progression of the disease [18–25].

The distribution of various lipids in the plasma membrane also appears to play a role in cell fusion, particularly the localization of the lipid phosphatidylserine (PS) [26–28]. It is usually found on the cytofacial site of the membrane and its asymmetrical localization is kept in a steady state by various membrane proteins, which can be divided into the classes of flippases/floppases (ATP-dependent P-type translocases, PS transport to the cytofacial side/exofacial side), and scramblases (ATP-independent distribution of PS to equilibrium) [29]. While in normal cells the exposure of PS functions acts as an “eat-me” signal in apoptotic cells, inducing non-inflammatory clearance, on cancer cells PS exposure functions as immunosuppressive signaling [30]. The PS exposure on cancer cells is, in contrast to apoptotic PS exposure, reversible, Ca^2+^-dependent and caspase 3/7 independent and is mainly caused by inhibition of flippases and activation of scramblases [31]. Anoctamin 6 (Ano6), also known as Transmembrane protein 16 F (TMEM16F), is a scramblase and distributes lipids mainly on the plasma membrane. It is one of the most highly expressed proteins in tissues and cells and functions additionally as a Ca^2+^-activated, non-selective ion-channel [29]. Ano6/TMEM16F plays a critical role in various physiological processes like necroptosis [32], pyroptosis [33] or ferroptosis [34, 35], trophoblast fusion and placental development [28, 36] as well as the activation of platelets [37]. Ano6/TMEM16F is also involved in the entry of viruses into cells by scrambling the host cells membrane [29].

In addition to scramblases, also flippases play a crucial role in the localization of phosphatidylserine in the plasma membrane. In the family of type IV P-type ATPases most exist as heterodimeric complex, whereby the catalytically active α-subunit consists of three cytoplasmic and ten transmembrane helices and the β-subunit functioning as chaperoning subunit, consists of two transmembrane helices and belongs to the cell cycle control protein 50 family (TMEM30, CDC50) [38, 39]. The both subunits are crucial for the correct folding and exit of the endoplasmic reticulum (ER) [40, 41]. In mammals, there are three isoforms of CDC50a proteins, CDC50a/TMEM30a, CDC50b/TMEM30b and TCDC50c/TMEM30c, of which CDC50a is the most highly expressed isoform interacting with most of the P4-ATPases [41]. The flippase Atp8a1 builds a heterodimer with the β-subunit CDC50a and was found mutated with single nucleotide polymorphism (SNPs) in metastatic breast cancer [42] and to play a role in cell migration by flipping phosphatidylethanolamine [43]. Additionally, there is a role of Atp8a1 in the promotion and progression of non-small cell lung cancer (NSCLC) [44] and it was found to be overexpressed in NSCLC tissue, where it promoted the expression of Vimentin, while suppressing E-Cadherin, promoting the invasion and migration ability of the cancer cells [45].

In order to characterize the influence of PS scrambling and localization in our breast cancer cell fusion model, we used different approaches to block PS on the cells’ surface, inhibit calcium-dependent scramblases or influence the PS localization by knocking out the scramblase Ano6/TMEM16F or overexpressing the flippase complex Atp8a1-CDC50a. We used the breast epithelial cell line M13SV1 in cocultures with the triple negative breast cancer cell lines MDA-MB-231 and HS578T in the dual split protein (DSP) assay, which we established and used in a previous study [46]. Since cell fusion between the cell lines does not happen spontaneously to a good quantifiable amount, the Syncytin-1 overexpression cell line M13SV1_Syn1 was used and effects on Syncytin-1-mediated cell fusion were studied. The results suggest that PS and PS-localizing proteins may have an influence on the Syncytin-1-mediated cell fusion of human breast epithelial cell with breast cancer cells.

## Methods

### Cell culture

M13SV1 human breast epithelial cells (kind gift of James Trosko, Michigan State University, East Lansing, MI, USA [47]), HS578T breast cancer cells (HTB 126; LGC Standards GmbH, Wesel, Germany), and MDA-MB-231 breast cancer cells (HTB 26; LGC Standards GmbH, Wesel, Germany) were maintained in standard media (MDA-MB-231: DMEM; HS578T and M13SV1: RPMI 1640) (PAN Biotech GmbH, Aidenbach, Germany) supplemented with 10% fetal calf serum (FCS; PAN Biotech GmbH, Aidenbach, Germany) and 100 U/ml penicillin/ 0.1 mg/ml streptomycin (PenStrep; PAN Biotech GmbH, Aidenbach, Germany). The following supplements were further added to the culture medium. M13SV1: 10 µg/ml recombinant human epidermal growth factor (rhEGF), 5 µg/ml human recombinant insulin, 0.5 µg/ml hydrocortisone, 4 µg/ml human transferrin, 10 nM β-estrogen (all supplements were purchased from Merck KGaA, Darmstadt, Germany). M13SV1 cells stably expressing pcDNA3.1_Syncytin-1-P2A-PuroR were already generated in the working group [46] and were maintained in medium with 2 µg/mL Puromycin (Thermo Fisher Scientific, Wesel, Germany).

Cell lines stably expressing DSP1-7 or DSP8-11 vector (MDA-MB-231_DSP1-7, HS578T_DSP1-7, M13SV1_DSP8-11, M13SV1_ASCT2KO_DSP8-11, M13SV1_Syn1_ DSP8-11) were already generated in the working group [46] and were maintained in their respective medium with additional 200 µg/ml Hygromycin B (Pan Biotech, Aidenbach, Germany). Cell lines stably expressing pcDNA3.1_Atp8a1-P2A-CDC50a-P2A-BSD vector were maintained in their respective medium with additional 25 µg/mL (M13SV1 cells) or 50 µg/mL (breast cancer cells) blasticidin (Gibco, Thermo Fisher Scientific, Wesel, Germany). All cells were cultivated in a humidified atmosphere at 37 °C and 5% CO_2_.

### Quantitative polymerase chain reaction (qPCR)

Total RNA was isolated from 1 × 10^6^ cells using the NucleoSpin^®^ RNA kit in accordance with the instruction manual (Macherey Nagel, Düren, Germany). The cDNA synthesis was performed using random hexamer primers and the First Strand cDNA Synthesis Kit (ThermoFisher Scientific GmbH, Schwerte, Germany) according to the manufacturer’s instructions. The qPCR was run on StepOnePlus™ Real-Time PCR System using 50 ng of the prepared cDNA and the Luna^®^ Universal qPCR Master Mix (New England Biolabs GmbH, Frankfurt am Main, Germany) as stated by the manufacturer. Specific primers (Thermo Fisher Scientific, Wesel, Germany) were used for the target genes Ano5/TMEM16E (fwd: 5′-GCG GCG GCT TAT GTT TCA AAA-3′; rev: 5′-CGC CTT TAA CTC TGC GTC TTT C-3′) [28], Ano6/TMEM16F (fwd: 5′-AAA TTG CCT CTG AAA CCC AAT GA-3′, rev: 5′-GCT TTC GTC TAC ACT GAG GAC TT-3′) [48], Ano7/TMEM16G (fwd: 5′-GAC CTG GCG GGA GAC TTT TC-3′, rev: 5′-CCC AGC CGC ACG AAG ATT AT-3′) [28] and GAPDH (fwd: 5′-tgc acc acc aac tgg ctt agc-3′,rev: 5′-ggc atg gac tgt ggt cat gag-3′). Initial denaturation was done for 1 min at 95 °C, followed by a two-step amplification phase of 45 cycles of 15 s at 95 °C and 30 s at 60 °C while sampling for fluorescence emission. Samples were run in triplicates. The qPCR data were analyzed using the StepOne software 2.3 (Thermo Fisher Scientific, Wesel, Germany) and the gene’s expression levels were calculated using the ∆CT method, Glyceraldehyde-3-Phosphate Dehydrogenase (GAPDH) was used as an internal control.

### Substances used to block/inhibit Ano6/TMEM16F activity

Based on the study by Braga et al. from 2021, the following substances were investigated for their effect on cell fusion between human breast epithelial cells and human breast cancer cells, as they showed both direct and indirect influence on Ano6/TMEM16F [26]: Niclosamide (Thermo Fisher Scientific, Wesel, Germany), Clofazimine (Thermo Fisher Scientific, Wesel, Germany), Salinomycin (Sigma Aldrich, Taufkirchen, Germany) and Hexachlorophene (Sigma Aldrich, Taufkirchen, Germany). All compounds were solubilized in dimethyl sulfoxide (DMSO) and used in the colony formation and proliferation assays in the following concentrations: Niclosamide (500 nM, 100 nM, 10 nM, 1 nM) Clofazimine (1 µM, 500 nM, 100 nM, 50 nM), Salinomycin (1 µM, 500 nM, 100 nM), Hexachlorophene (1 µM, 500 nM, 100 nM). DMSO (1% (v/v)) was used as a control.

### Generation of Ano6 knock-out (KO) cell lines

CRISPR/Cas9 was used to achieve an Ano6/TMEM16F KO in M13SV1_DSP8-11, MDA-MB-231_DSP1-7 and HS578T_DSP1-7 cells. The guide RNA sequence (5′-AAT AGT ACT CAC AAA CTC CG -3′) published by Zhang et al. with the according sense and antisense oligonucleotides (gRNA1_ANO6_fwd: 5′-CAC CAA TAG TAC TCA CAA ACT CCG-3′, gRNA1_ANO6_rev: 5′-AAA CCG GAG TTT GTG AGT ACT ATT-3′) was used for the generation of a CRISPR/Cas9 all-in vector [28]. The oligonucleotides were annealed and ligated into the BbSI digested pX330-U6-Chimeric_BB-CBh-hSpCas9-P2A-PuroR vector, the production of which is described elsewhere [49]. Cloning was verified by Sanger Sequencing (Eurofins Genomics, Ebersbach, Germany). The sequences were analyzed using SnapGene 5.3.1 software (Dotmatics, Boston, MA, USA).

M13SV1_DSP8-11, MDA-MB-231_DSP1-7 and HS578T_DSP1-7 cells were transfected with pX330-U6-Chimeric_BB-CBh-hSpCas9-P2A-PuroR_Ano6_1 (1 µg) using the jetOPTIMUS^®^ DNA transfection reagent as recommended by the manufacturer (Sartorius Lab Instruments, Göttingen, Germany). To select transfected cells from non-transfected cells, 2 µg/mL Puromycin (Thermo Fisher Scientific, Wesel, Germany) was added to the culture medium 24 h after transfection for 48 h. Dead cells were removed by washing with phosphate buffered saline (PBS). For the isolation of single cell clones, 200 cells were seeded in a 100 mm cell culture dish. Single-cell clones were isolated using self-punched cloning discs made from Rotilabo blotting paper (0.35 mm, Roth, Karlsruhe, Germany) with a diameter of approximately 3 mm, were then transferred to 24 well plates and propagated. Growing clones were transferred to bigger cell culture flasks once they reached confluence.

The successful KO of Ano6 was validated by Sanger Sequencing. Therefore, genomic DNA of the KO cell lines was isolated using the NucleoSpin^®^ Tissue kit in accordance with the manufacturer’s instructions (Macherey Nagel GmbH, Düren, Germany). The gene fragment edited by CRISPR/Cas9 was amplified by PCR (Primers: Seq_Ano6_1_fwd: 5′-CAG AAA CAC TTG TTC CCA TCC-3′, Seq_Ano6_1_rev: 5′-ACA TTT GCT TGC TGT GTA CC-3′) using the Q5^®^ Hot Start High Fidelity DNA Polymerase (New England Biolabs, Frankfurt am Main, Germany) and then Sanger Sequencing (Eurofins Genomics, Ebersbach. Germany) was done, using the primer SeqPrimer_Ano6_1_fwd (5′-GTG AAC TCC CAT TTG CAA TTA C-3′). Analysis of sequencing data was done using SnapGene 5.3.1 software (Dotmatics, Boston, MA, USA). Only single cell clones with a stable KO of Ano6 were pooled to receive a heterogeneous cell population with a homogenous Ano6 KO and were used for the following studies.

### Generation of Atp8a1 overexpressing cell lines

A vector coding for an Atp8a1-P2A-CDC50a-P2A-BSD sequence for simultaneous and stable Atp8a1 and CDC50a overexpression was constructed using the In Fusion^®^ HD Cloning Kit (Takara, Saint Germain en Laye, France). Briefly, pcDNA3.1_Atp8a1-P2A-BSD was linearized by PCR (IF_Atp8a1-CDC50-BSD_BSD_fwd: 5′-ATG CAT GCC AAG CCT TTG TCT-3′, IF_Atp8a1-CDC50-BSD_Atp8a1_rev: 5′-AGG GCC GGG ATT CTC CTC-3′). The CDC50a fragment was amplified by PCR from pcDNAintron-CDC50A-c3xFLAG (a gift from Melinda Brindley; Addgene plasmid #215702; http://n2t.net/addgene:215702; RRID: Addgene_215702; IF_Atp8a1-CDC50-BSD_CDC50_fwd: 5′-TGG AGG AGA ATC CCG GCC CTA TGG CGA TGA ACT ATA ACG CGA AG-3′, IF_Atp8a1-CDC50-BSD_CDC_rev short: 5′-CAT CGT CAT CCT TGT AGT CGA TAT C-3′). In order to create a P2A side between the CDC50a fragment and the blasticidin resistance gene, a short double stranded DNA fragment was received by heating two complementary single stranded oligos (IF_Oligo new P2A_fwd: 5′-ACA AGG ATG ACG ATG GCA GCG GCG CCA CAA ACT TCT CTC TGC TAA AGC AAG CAG GTG ATG TTG AAG AAA ACC CCG GGC CT-3′, IF_Oligo new P2A_rev: 5′-AGG CTT GGC ATG CAT AGG CCC GGG GTT TTC TTC AAC ATC ACC TGC TTG CTT TAG CAG AGA GAA GTT TGT GGC GCC GCT GC-3′) up to 95 °C and slowly cooling down to room temperature, resulting in single stranded DNA fragment with overhangs needed for In-Fusion^®^ Cloning. The cloning reaction was set up as suggested by the manufacturer by using a molecular ratio of linearized vector: CDC50a fragment: P2A oligo fragment of 1:2:15. The sequence of the resulting vector pcDNA3.1_Atp8a1-P2A-CDC50a-P2A-BSD was verified by Sanger sequencing (Eurofins Genomics, Ebersbach. Germany; Seq_Atp8a13069_fwd: 5′-TCA GGA GAG GCA GCC ATG TTG TTC-3′, Seq_BSD_rev: 5′-GAC GCT GTA GTC TTC AGA GAT GGG-3′).

M13SV1_DSP8-11, M13SV1_Syncytin-1_DSP8-11, MDA-MB-231_DSP1-7 and HS578T_DSP1-7 cells were transfected with pcDNA3.1_Atp8a1-P2A-CDC50a-P2A-BSD (2 µg) using the jetOPTIMUS^®^ DNA transfection reagent as recommended by the manufacturer (Sartorius Lab Instruments, Göttingen, Germany). To select transfected cells from non-transfected cells, 25 µg/mL (M13SV1 cells) or 50 µg/mL (breast cancer cells) blasticidin (Gibco, Thermo Fisher Scientific, Wesel, Germany) was added continuously to the culture medium 24 h after transfection.

### Dual split protein assay

For the quantification of cell fusion, the dual split protein (DSP) assay was used, which is described elsewhere [46]. For the experiments of heterogenic cell fusion with the different Ano6 inhibiting substances, the cell lines were set up in a ratio breast epithelial cell line: breast cancer cell line of 1:3 in a 96-well plate in duplicates in the respective medium of the breast cancer cell line. Niclosamide, Clofazimine, Salinomycin and Hexachlorophene were individually added to the media to reach final concentration of either 100 nM or 500 nM. Untreated cells were used as controls, as well as cells whose medium was mixed with 1% (v/v) DMSO to exclude possible effects of the solvent on cell fusion. The cells were incubated for 48 h in a humidified atmosphere at 37 °C and 5% CO_2_. Cocultured cells were harvested, washed once with PBS and the amount of green fluorescent cells was quantified by flow cytometry (FACSCalibur™, Becton Dickenson, Heidelberg, Germany). As negative control and for defining a threshold, freshly mixed cells were measured.

For the experiments of heterogenic cell fusion, the different genetically altered cell lines were set in a ratio breast epithelial cell line: breast cancer cell line of 1:3 in a 96-well plate in triplicates in the respective medium of the breast cancer cell line. For masking PS in the assay, 0.25 µg/mL Annexin V (CF^®^568 Annexin V, Biotium, Fremont, CA, USA) was added to the coculture medium. The cells were incubated for 72 h in a humidified atmosphere at 37 °C and 5% CO_2_. Cocultured cells were harvested, washed once with PBS and the amount of green fluorescent cells was quantified by flow cytometry (CytoFLEX, Beckman Coulter Life Sciences, Krefeld, Germany). As negative control and for defining a threshold, freshly mixed cells were measured. In the following results and discussion sections, the labeling of the cell lines with DSP vectors has been omitted for easier readability.

### Flow cytometry

The amount of green fluorescent cells in Ano6 inhibition cell fusion assays was determined by flow cytometry using a FACSCalibur™ flow cytometer (Becton Dickenson, Heidelberg, Germany). The cocultured and treated cells were harvested, washed with PBS once and resuspended in PBS. The flow cytometry data were analyzed using WinMDI 2.8 (https://winmid.software.informer.com/2.8/ accessed on February 1st, 2020).

The amount of green fluorescent cells in cell fusion assays with genetically modified cell lines as well as the AnxV inhibition assay was determined by flow cytometry using a CytoFLEX flow cytometer (Beckman Coulter Life Sciences, Krefeld, Germany). The cocultured cells were harvested, washed with PBS once and resuspended in PBS. The flow cytometry data were analyzed using CytExpert software (Version 2.4.0.28, Beckman Coulter, Inc., Krefeld, Germany).

### Phosphatidylserine externalization assay

The amount of cells with externalized PS was assessed by using Fluorescein isothiocyanate (FITC) coupled Annexin V (Anx V). Therefore, 7,500 cells were seeded in 96-well plates and were incubated until the cells were adherent. Thereafter, the media was replaced with fresh media containing the added substances in the different concentrations stated in Sect.  4.2. and additionally 10 µM Ionomycin was used as a positive control on the cells, which can activate Ano6 by enhancing the intracellular calcium concentration. The cells were incubated for one hour and afterwards were treated with AnxV-FITC in 1× binding buffer (Miltenyi Biotec BV & Co. KG, Bergisch Gladbach, Germany) as stated by the manufacturer. Five random sections of each well were recorded using a fluorescence microscope (Leica DM IL, Leica Microsystems GmbH, Wetzlar, Germany), and the number of green fluorescent and thus PS-positive cells was counted manually using ImageJ (Image J; https://Fiji.sc, Version 1.54f).

### Western blot analysis

Cell lysates for Western Blots were prepared differently, depending on which target protein was assayed. For blotting β-Actin and Flag, as well as the used epithelial-to-mesenchymal transition (EMT) markers, cells were harvested, washed once with PBS, adjusted to a cell number of 2∙10^5^ cells and lysed with 3× Laemmli sample buffer (10 min, 95 °C).

For blotting of Ano6/TMEM16F, 1 × 10^6^ cells were pelleted by centrifugation for 5 min at 1000 × g at 4 °C. The pellet was washed with PBS once and 100 µL RIPA buffer (Pierce^®^, Thermo Scientific, Wesel, Germany) with protease/phosphatase inhibitor (Cell Signaling Technology, Leiden, the Netherlands) were added. The sample was vortexed and kept on ice for 30 min with occasional vortexing. Afterwards, the sample was sonicated three times for 10 s with 10 s pause on ice with a 40% amplitude (UP50H, Hielscher Ultrasonics GmbH, Teltow, Germany). The sample was centrifuged at 10,000 g for 20 min at 4 °C to pellet the debris and the supernatant was transferred to a fresh tube. The protein concentration was determined using the Pierce™ BCA Protein Assay Kit (Thermo Scientific, Wesel, Germany) and samples were adjusted to the same concentration with PBS. 3× Laemmli sample buffer was added and the samples were stored at -20 °C until usage.

For blotting of Atp8a1, up to 5 × 10^6^ cells were used for the purification of the membrane proteins using the Mem-PER™ Plus Membrane Protein Extraction Kit (Thermo Scientific, Wesel, Germany) as specified by the manufacturer. The protein concentration was determined using the Pierce™ BCA Protein Assay Kit (Thermo Scientific, Wesel, Germany) and samples were adjusted to the same concentration with PBS. 3× Laemmli sample buffer was added and the samples were stored at -20 °C until usage.

The samples were loaded on discontinuous polyacrylamide gels (8–10%) and proteins were separated by sodium dodecyl sulfate polyacrylamide gel electrophoresis (SDS-PAGE). Afterwards, the proteins were transferred to a nitrocellulose blotting membrane (Amersham Protran 0.45 μm NC, Cytiva, Freiburg im Breisgau, Germany) under semi dry conditions. The membranes were blocked with 5% (w/v) non-fat milk powder in Tris buffered saline with 1% (v/v) Tween 20 (TBS-T). The following antibodies were used in this study: β-Actin (Cat. No. A5316, clone AC-74, mouse mAb; Sigma Aldrich, Taufkirchen, Germany), Ano6/TMEM16F (Cat. No. PA5-58610, Invitrogen™ rabbit pAb, Thermo Fisher Scientific, Wesel, Germany), Atp8a1 (Cat. No. 21565-1-AP, rabbit pAb, Chromotek & Proteintech Germany, Planegg-Martinsried, Germany), Flag (Cat. No. 20543-1-AP, rabbit pAb, Chromotek & Proteintech Germany, Planegg-Martinsried, Germany), E-Cadherin (Cat. No. 3195, clone 24E10, rabbit mAb, Cell Signaling Technology, Frankfurt am Main, Germany), N-Cadherin (Cat. No. 14215, clone 13A9, mouse mAb, Cell Signaling Technology, Frankfurt am Main, Germany), Snail (Cat. No. 3879, clone C15D3, rabbit mAb, Cell Signaling Technology, Frankfurt am Main, Germany), Vimentin (Cat. No. 3932, rabbit pAb, Cell Signaling Technology), ZEB1 (Cat. No. 3396, clone D808D3, rabbit mAb, Cell Signaling Technology, Frankfurt am Main, Germany).

Secondary antibodies were coupled to horse radish peroxidase (Cell Signaling Technology, Frankfurt am Main, Germany). The antibody dilutions used in this study were in accordance with the manufacturer’s instructions. Bands were visualized using the Pierce™ ECL Western Blot substrate (Thermo Scientific, Wesel, Germany) and the Aequoria Macroscopic Imaging System (Hamamatsu Photonics Germany, Herrsching am Ammersee, Germany).

### Colony formation assay

Cells were seeded in 6-well plates (200 cells/well; Sarstedt AG & Co KG, Nümbrecht, Germany) were cultivated in complete media containing the compounds in the different concentrations stated for ten days in a humidified atmosphere at 37° C and 5% CO_2_. Thereafter, the media was removed, cells were washed twice with PBS, were then fixed with 4% paraformaldehyde and stained with 0.5% crystal violet (Merck KGaA, Darmstadt, Germany) for 30 min at room temperature. Plates were thoroughly washed with water and air-dried. The plates with the stained cell colonies were scanned and the images obtained were analyzed using Fiji software (Image J; https://Fiji.sc, Version 1.54f).

### Ano6 sequencing

Total RNA was isolated from 1 × 10^6^ cells using the NucleoSpin^®^ RNA kit in accordance with the instruction manual (Macherey Nagel, Düren, Germany). The cDNA synthesis was performed using random hexamer primers and the Maxima H Minus First Strand cDNA Synthesis Kit (ThermoFisher Scientific GmbH, Schwerte, Germany) according to the manufacturer’s instructions. For the subsequent sequencing, three fragments of the Ano6 mRNA were first amplified by PCR. The following primers (Thermo Fisher Scientific, Wesel, Germany) were used for this purpose: Fragment 1: fwd: 5’- TCG CGC CAA GTT CGG AG-3’, rev: 5’-CTG CAA AGA CCA GGG TTC CA-3’; Fragment 2: fwd: 5’- ACA AGG CAG CTT TCC CAC TC-3’, rev: 5’-ACC AGG CGT CTA AAC TGG GTG G-3’; Fragment 3: fwd: 5’-GAG GTG TTA TGT ACG TAT GC-3’, rev: 5’-CTT TCT GGA GGT ATT GAA AAT AC-3’. All fragments were amplified by PCR (annealing temperature 60 °C, elongation time 3 min, 72 °C; 35 cycles) using the Q5^®^ Hot Start High Fidelity DNA Polymerase (New England Biolabs, Frankfurt am Main, Germany). Subsequently, the PCR products were loaded on a 1% Agarose gel and PCR fragments were isolated using the Gel and PCR Clean-Up Kit (Macherey Nagel, Düren, Germany) as referred to the instruction manual. The isolated PCR fragments were then analyzed by Sanger Sequencing (Eurofins Genomics, Ebersbach. Germany) using the following sequencing primers (Thermo Fisher Scientific, Wesel, Germany): Sequencing primer Fragment 1 fwd: 5’-TTT GCT ACA AAT GGA GGA GGA GG-3’; Sequencing primer Fragment 1 rev: 5’-AAA CTC TTG CTC TGG CTT GAT G-3’; Sequencing primer Fragment 2 fwd: 5’-ACA AGG CAG CTT TCC CAC TC-3’; Sequencing primer Fragment 2 rev: 5’-AAA TAA TCC CAG TTT GCC CAT AGG-3’; Sequencing primer Fragment 3 fwd1: 5’-TAT TGG AAA TAA GAG TGG ACG C-3’; Sequencing primer Fragment 3 fwd2: TGA AGA GTG TGA CCC AGG TG-3’; Sequencing primer Fragment 3 rev: 5’-AGA TGA CCC TGG AAC AGA TCT AGC-3’. Analysis of sequencing data was done using SnapGene^®^ 5.3.1 software (Dotmatics, Boston, MA, USA).

### Statistical analysis

Statistical analysis was performed using GraphPad PRISM 9.5 (https://graphpad.com). A detailed description of which statistical test was used is given in the appropriate figure legends.

## Results

For better readability, the additions DSP1-7 and DSP8-11 have been omitted. However, it should be noted that all cell lines expressed these stably. DSP1-7 was expressed stably by MDA-MB-231 and HS578T cells, while DSP8-11 was expressed in all genetically modified M13SV1 cell lines used.

### Masking of phosphatidylserine with Annexin V leads to a significant reduction of fused cells in cocultures of M13SV1_Syn1 and MDA-MB-231 cells

**Fig. 1:**
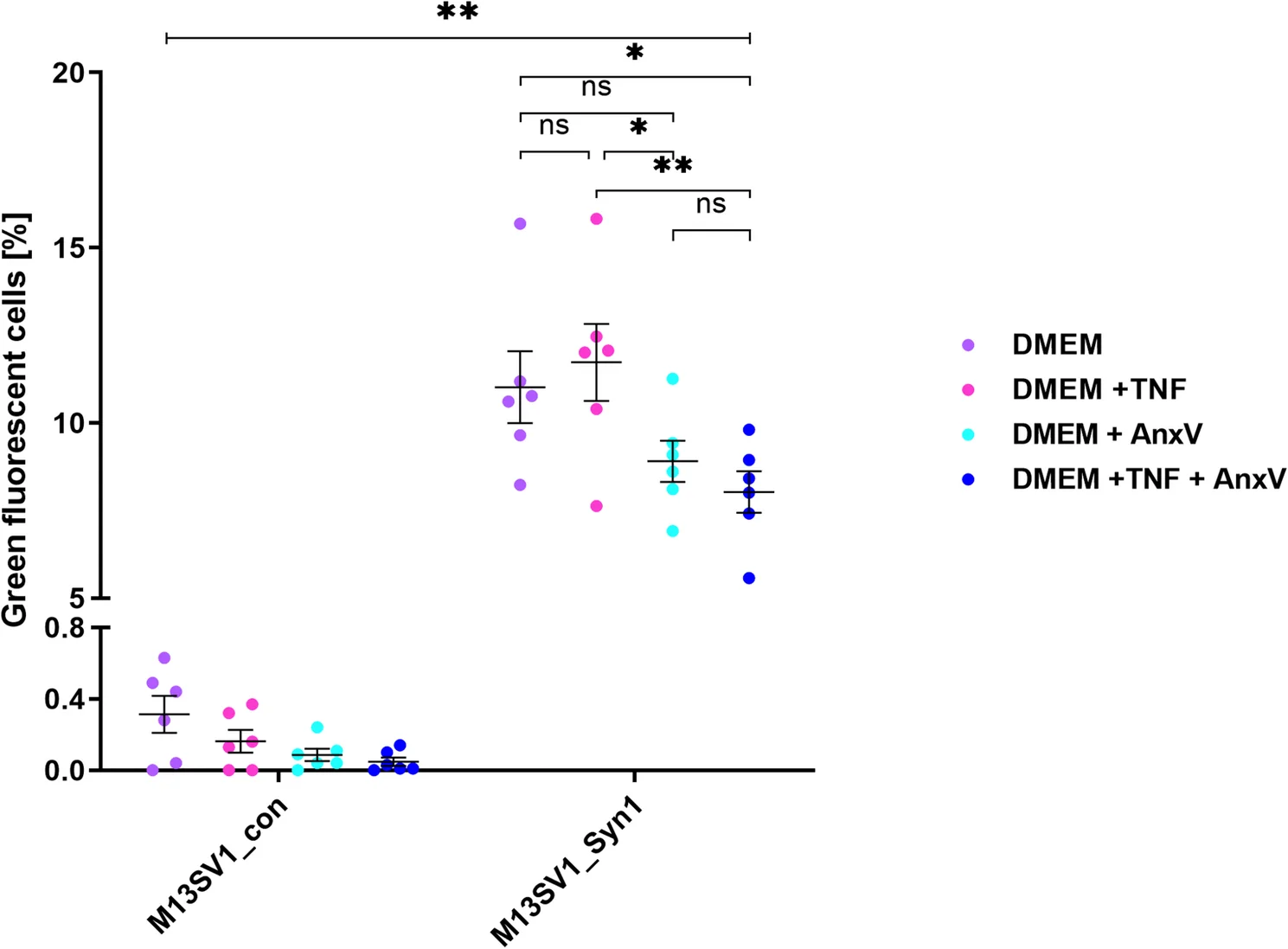
Fusion of M13SV1 breast epithelial cells and MDA-MB-231 breast cancer cells is influenced by the addition of Annexin V (AnxV)**.** M13SV1 cells were used as control cells (M13SV1_con) or as cells overexpressing Syncytin-1 (M13SV1_Syn1). The cocultures were set up in a ratio of 1:3 for 72 h in DMEM, DMEM with 100 ng/mL TNFα, DMEM with 0.25 µg/mL AnxV or DMEM containing both TNFα and AnxV. The amount of green fluorescent cells was measured by flow cytometry. Shown are the means ± S.E.M. of six independent experiments. Statistical significance was calculated using a two-way ANOVA and Tukey’s multiple comparison test. The upper bar shows the significant difference in cell fusion of the cocultures with M13SV1_Syn1 cells in comparison to M13SV1_con cells. The lower bars show the effects of the medium used on Syncytin-1-mediated cell fusion. *p* > 0.05 = ns; *p* ≤ 0.05 = *; *p* ≤ 0.01 = **

The phospholipid PS has been suggested as cell fusion relevant molecule [27]. To prove whether PS may also be important for the merger of human breast epithelial cells and human breast cancer cells, we studied the fusion frequency of control and Syncytin-1 overexpressing M13SV1 breast epithelial cells (M13SV1_con, M13SV1_Syn1) and the breast cancer cell line MDA-MB-231 cells by using the DSP cell fusion assay, since the coculturing of these cells showed their high fusogenicity in previous studies [46], and masking PS with Annexin V (AnxV). AnxV is a 35 kDa protein and belongs to a family of ubiquitous eukaryotic cytoplasmic proteins, which bind Ca^2+^ and interact with phospholipids [50]. Additionally, to mimic an inflammatory environment as it occurs in and around tumors, the inflammatory cytokine TNFα was added to the coculture medium. Cocultures were set up for 72 h in the specific media and the amount of green fluorescent cells was quantified by flow cytometry (Fig. 1). As seen in previous studies, the cell fusion between M13SV1_con cells and MDA-MB-231 cells was rather minor [46]. In comparison to this, in the coculture of M13SV1_Syn1 cells with MDA-MB-231 cells, the amount of green fluorescent cells was significantly increased (11.03 ± 1.03%; Fig. 1), but also effects of the different media could be observed in the Syncytin-1-mediated cell fusion. In line with our previous study [46], the addition of TNFα in the coculture medium led to a slight increase in fluorescent cells (11.74 ± 1.10%; Fig. 1), therefore increasing the cell fusion rate. In contrast, the addition of AnxV slightly decreased the amount of green fluorescent cells (8.91 ± 0.59%; Fig. 1), which was further significantly decreased when AnxV and TNFα were used simultaneously (8.03 ± 0.59%; Fig. 1).

### Ano6/TMEM16F is the most highly expressed scramblase in the cell lines used

**Fig. 2:**
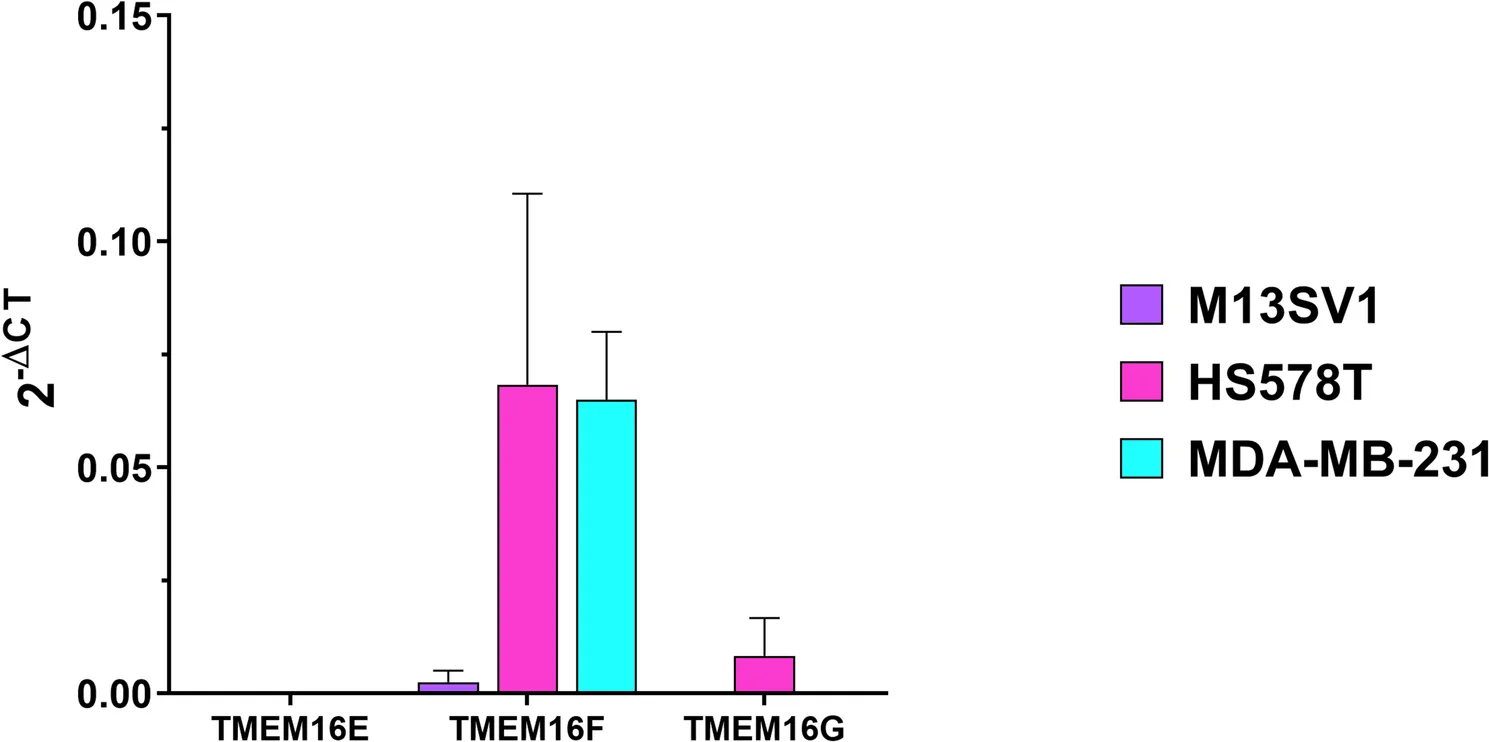
Ano6/TMEM16F is highly expressed in HS578T and MDA-MB-231 breast cancer cells. The relative expression of Ano5/TMEM16E, Ano6/TMEM16F and Ano7/TMEM16G in M13SV1, HS578T and MDA-MB-231 cells was determined by qPCR in relation to the housekeeping gene GAPDH. Shown are the means ± S.E.M. of two to six independent experiments. Data were not significant

In order to analyze whether the effect of masking PS with AnxV on cell fusion is related to the scrambling of PS in the plasma membrane, gene expression of the scramblases Ano5/TMEM16E, Ano6/TMEM16F and Ano7/TMEM16G was examined using qPCR (Fig. 2). The qPCR analysis shows that Ano6/TMEM16F is the most prominent expressed TMEM family member in the used cell lines, while the breast cancer cell lines HS578T and MDA-MB-231 express it higher than the breast epithelial cell line M13SV1. HS578T is the only cell line which also expresses another scramblase, Ano7/TMEM16G, but rather low in comparison to Ano6/TMEM16F (Fig. 2).

### Direct or indirect inhibition of Ano6/TMEM16F by clofazimine, niclosamide, salinomycin or hexachlorophene has mostly no significant effect on PS externalization

Braga et al. demonstrated that the Ano6/TMEM16F activity of cells could be indirectly and directly inhibited by specific drugs, such as Clofazimine, Niclosamide, Salinomycin and Hexachlorophene, which all resulted in lower levels of externalized PS [26]. Therefore, the M13SV1-Syn1, HS578T and MDA-MB-231 cells were incubated with different concentrations of these drugs and the relative amount of PS positive cells was determined by Annexin V-FITC staining.

**Fig. 3:**
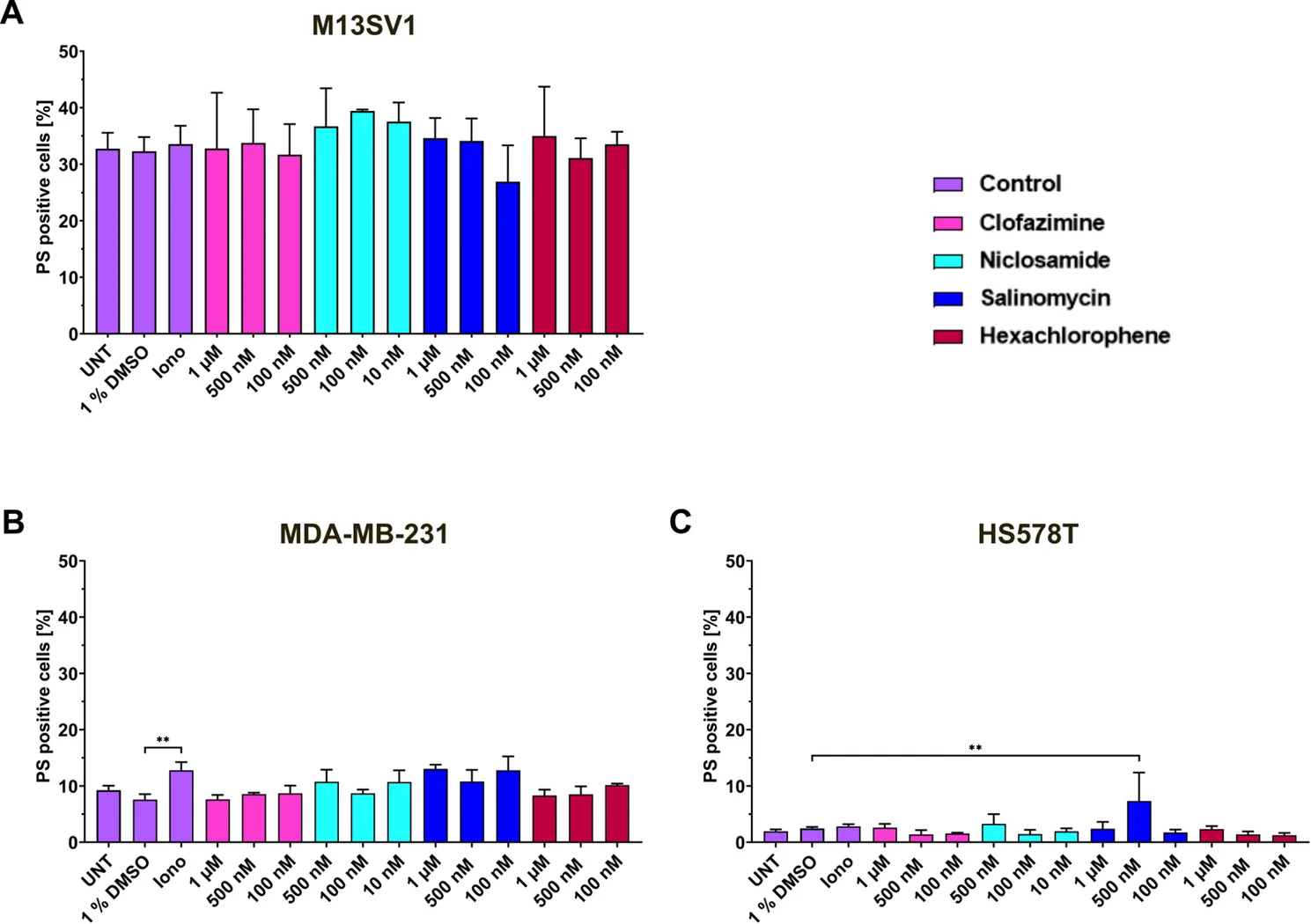
Inhibition of Ano6/TMEM16F activity did not result in reduced PS externalization levels**.** Ionomycin (Iono) was used as a positive control for Ano6/TMEM16F activation (10 µM). Analysis was done without additives (UNT), with 1% DMSO as a vehicle control, or with different concentrations of the substances Clofazimine, Niclosamide, Salinomycin or Hexachlorophene. Fluorescence microscopy pictures were taken and the amount of all cells was set in ratio to the amount of green fluorescent cells by counting for (**A**) M13SV1_Syn1, (**B**) MDA-MB-231 and (**C**) HS578T cells. Shown are the means ± S.E.M. of three independent experiments. Statistical analysis was done using a Kruskal-Wallis nonparametric test and Dunnett’s multiple comparison post hoc test. *p* ≤ 0.01 = **

The cells did not react to the vehicle control 1% DMSO with a change of PS exposed on the cell surface in comparison to untreated cells (UNT) (M13SV1_Syn1: UNT 32.76 ± 2.80% vs. 1% DMSO 32.30 ± 2.49%; MDA-MB-231: UNT 9.27 ± 0.79% vs. 1% DMSO 7.59 ± 0.99%; HS578T: UNT 1.94 ± 0.35% vs. 1% DMSO 2.47 ± 0.25%; Fig. 3A-C). The four substances used did not show any significant effects on the cell lines, except for 500 nM Salinomycin on HS578T cells, which significantly increased the percentage of cells exposing PS on their surface (7.33 ± 5.05%; Fig. 3C).

### Direct or indirect inhibition of Ano6 by clofazimine, niclosamide, salinomycin or hexachlorophene has no significant effect on the Syncytin-1-mediated cell fusion

**Fig. 4:**
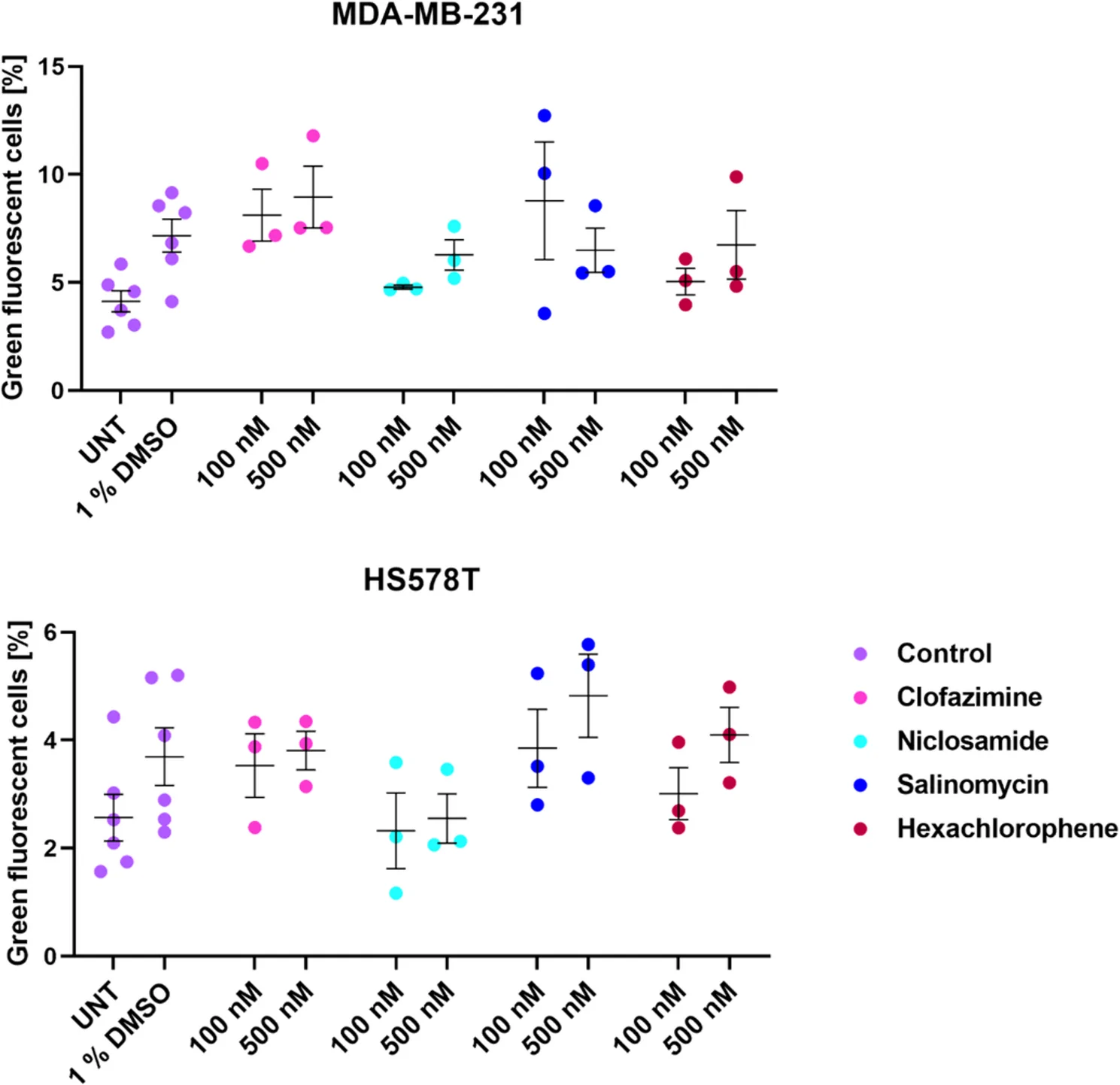
The relative fusion rate of M13SV1_Syn1 cells with MDA-MB-231 cells or HS578T cells was not impaired by influencing PS externalization.** (A)** MDA-MB-231, **(B)** HS578T. The medium was used either without further additives (UNT), with 1% DMSO as a vehicle control, or with 100 nM or 500 nM of the substances Clofazimine, Niclosamide, Salinomycin or Hexachlorophene. The amount of green fluorescent cells was measured by flow cytometry. Shown are the means ± S.E.M. of three independent experiments. Statistical significance of 1%DMSO control vs. untreated cells (UNT) and the substances was calculated using a two-way ANOVA and Tukey’s multiple comparison test; all comparisons are not significant

To test the effect of direct or indirect inhibition of Ano6/TMEM16F on the cell fusion between M13SV1_Syn1 cells with MDA-MB-231 or HS578T cells, the DSP assay was set up by coculturing the cells for 48 h with the different substances in two different concentrations. The amount of green fluorescent cells was measured by flow cytometry and compared to the vehicle control cells treated with 1% DMSO (Fig. 4). The results of the cell fusion assays show that 1% DMSO as vehicle control for the substances was able to slightly increase the amount of green fluorescent cells in comparison to the untreated control cells (MDA-MB-231 UNT: 4.13 ± 0.49%; 1% DMSO: 7.17 ± 0.76%; HS578T UNT: 2.57 ± 0.43%; 1% DMSO: 3.69 ± 0.53%; Fig. 4). The addition of the different substances had no significant effect for both cancer cell lines, but a slight, not significant decrease in cell fusion was seen for cocultures treated with Niclosamide (MDA-MB-231 100 nM: 4.78 ± 0.09%; 500 nM: 6.28 ± 0.70%; HS578T 100 nM: 2.32 ± 0.70%; 500 nM: 2.55 ± 0.46%; Fig. 4) and 100 nM Hexachlorophene (MDA-MB-231: 5.05 ± 0.61%; HS578T: 3.01 ± 0.48%; Fig. 4) in comparison to the 1% DMSO control.

### A CRISPR/Cas9-mediated KO of Ano6/TMEM16F shows no significant effect on Syncytin-1-mediated cancer cell fusion

**Fig. 5:**
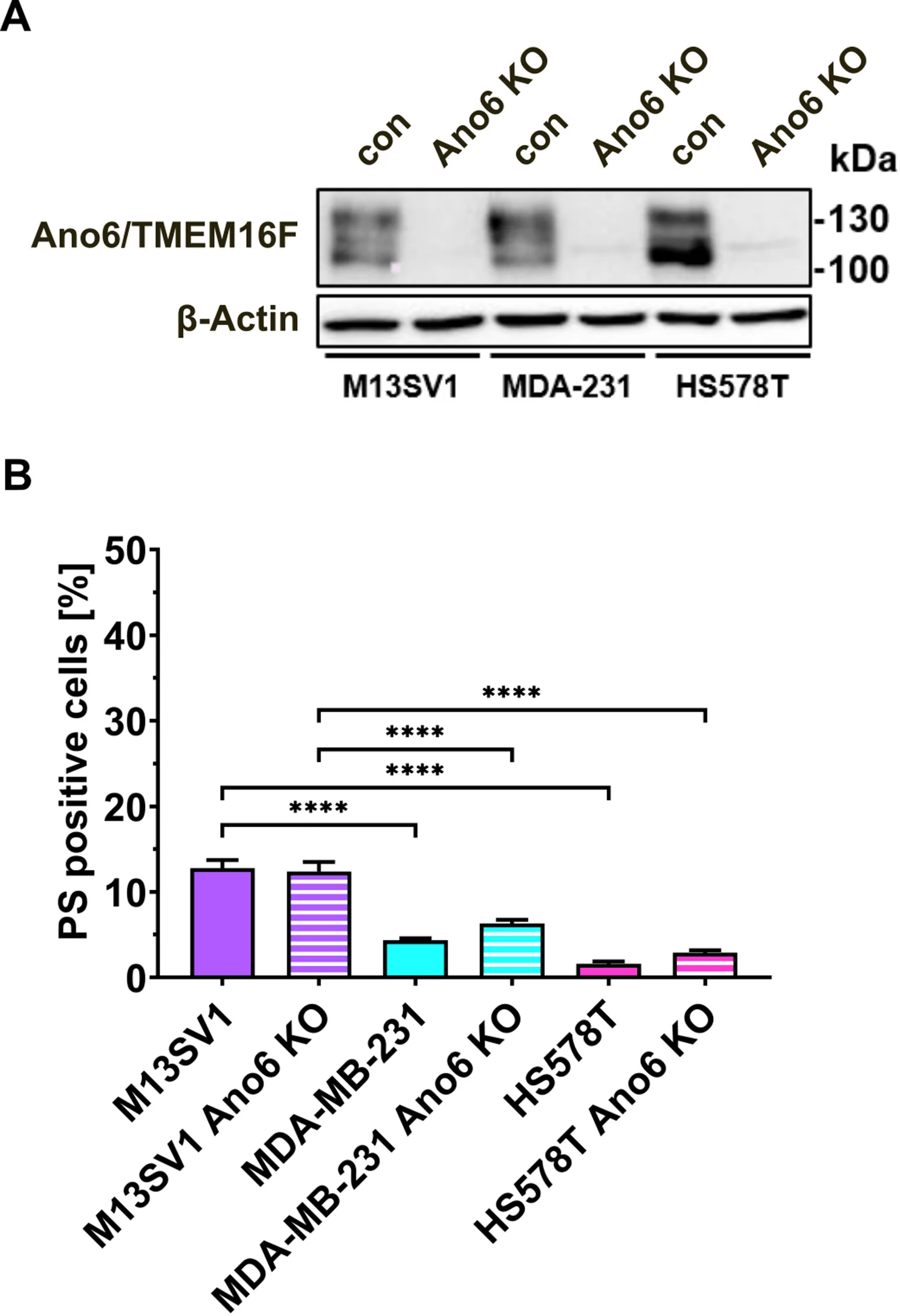
Successful CRISPR/Cas9 mediated Ano6/TMEM16F knock-out in M13SV1, MDA-MB-231 and HS578T cells. **(A)** β-Actin was used as loading control. Shown are representative Western blots of at least three independent experiments. (**B**) The Ano6/TMEM16F KO had no effect on the cells’ PS externalization levels. Fluorescence microscopy pictures were taken and the amount of all cells was set in ratio to the amount of green fluorescent cells. Shown are the means ± S.E.M. of at least three independent experiments. Statistical analysis was done using an ordinary one-way ANOVA and a Tukey multiple comparisons post-hoc test. *p* ≤ 0.001 = ****. Data with Ionomycin as a positive control (10 µM) are summarized in SI Figure S1

**Fig. 6:**
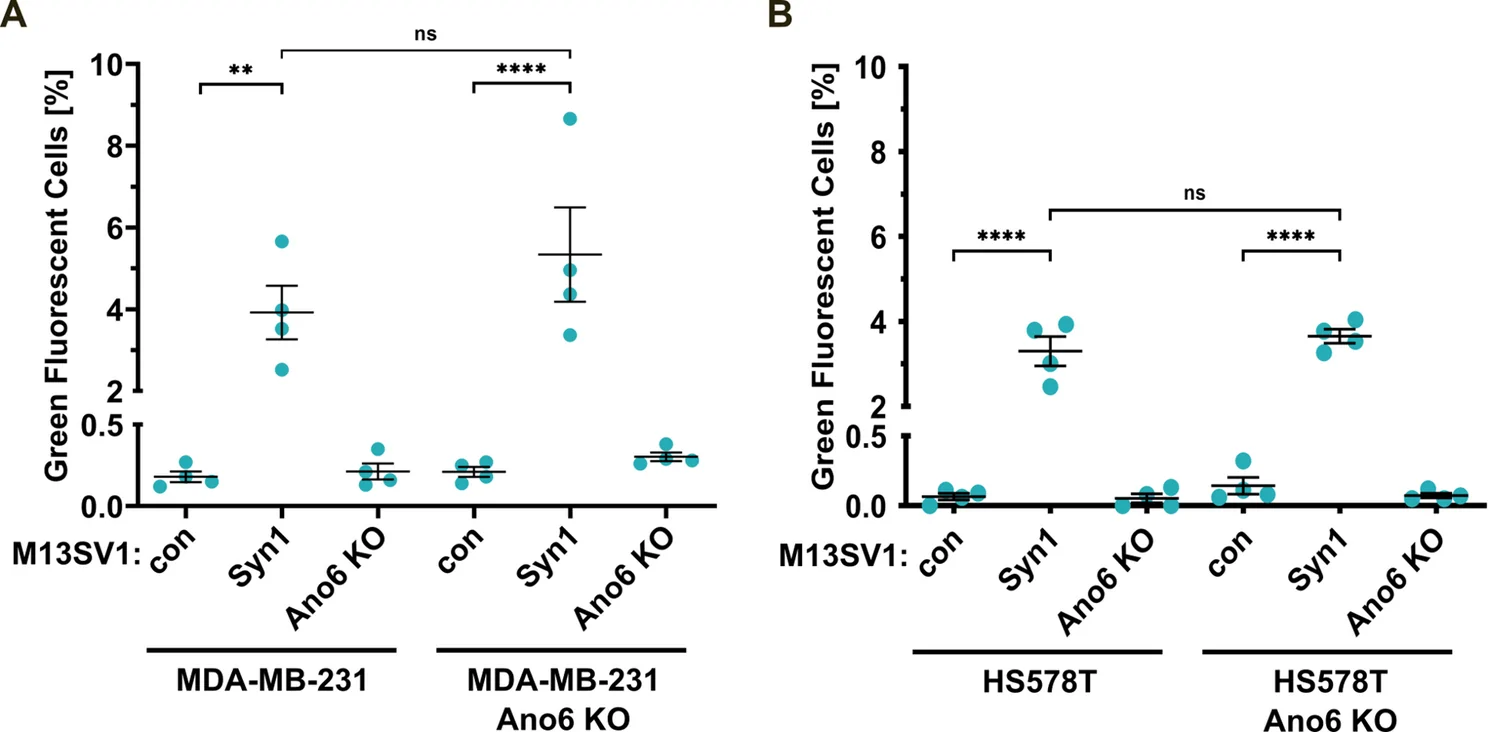
The relative cell-cell fusion rate of M13SV1 and MDA-MB-231 and HS578T cells remained unchanged after Ano6/TMEM16F knock-out**. (A)** MDA-MB-231, **(B)** HS578T breast cancer cell lines. The amount of green fluorescent cells was measured by flow cytometry. Shown are the means ± S.E.M. of four independent experiments. Statistical significance was calculated using a one-way ANOVA and Bonferroni’s multiple comparison test. *p* ≤ 0.01 = **; *p* ≤ 0.001 = ***

To further analyze the role of Ano6/TMEM16F in cell fusion between the used cell lines, a CRISPR/Cas9-mediated KO of Ano6/TMEM16F was achieved in M13SV1, MDA-MB-231 and HS578T cells. Therefore, the cells were transfected with the CRISPR/Cas9 vector, then single cell clones were isolated and analyzed by Western Blot and sequencing (Fig. 5A). Three to four single cell clones bearing the KO of each cell line were pooled and used as Ano6/TMEM16F KO (Ano6 KO) cell line for further experiments. The Ano6/TMEM16F KO had no effect on the cells’ PS externalization capacity (Fig. 5B). A heterotypic cell fusion assay was set up with the three M13SV1 cell lines, either combining the control cell line (con), the Syncytin-1 overexpressing cell line (Syn1) or the Ano6 KO cell line (KO), with the MDA-MB-231 or HS578T cell line as control cell line or bearing the Ano6 KO in a ratio of 1:3 for 72 h. The amount of green fluorescent cells was subsequently measured with flow cytometry (Fig. 6A, B). The results of the DSP assays with the breast cancer cell lines as control cells or bearing an ANO6 KO show that cocultures with M13SV1 wild control (con) or Ano6 KO cells (Ano6 KO) yielded in negligible amounts of green fluorescent cells. The coculturing with M13SV1 cells overexpressing Syncytin-1 (Syn1) led to increased amounts of green fluorescent cells, independent of the Ano6 KO in the breast cancer cell lines (Fig. 6A, B). When comparing the values for Syncytin-1 mediated cell fusion with the normal breast cancer cell lines and the breast cancer cell lines bearing the Ano6 KO, there was a slight increase in green fluorescent cells for the Ano6 KO breast cancer cell lines (Fig. 6A, B). This indicates that Ano6/TMEM16F is likely not required for the breast cancer cell lines to fuse with the Syncytin-1 overexpressing M13SV1 cells.

### Ano6 sequencing

**Fig. 7:**
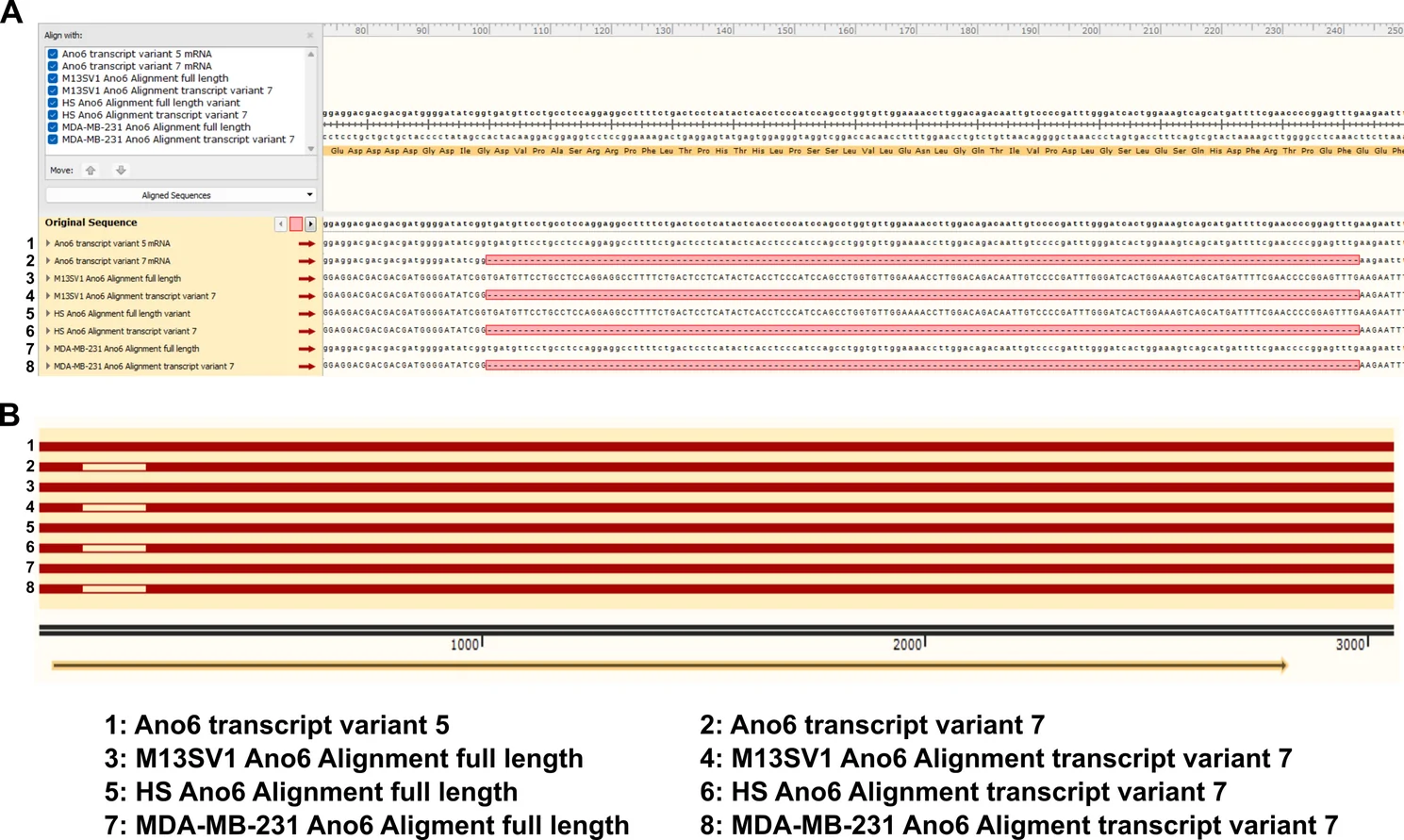
Two Ano6/TMEM16F transcript variants are expressed in M13SV1, MDA-MB-231 and HS578T cells. **(A)** The obtained sequencing results were aligned to the Ano6/TMEM16F transcript variants 5 and 7. Compared to Ano6/TMEM16F transcript variant 5, transcript variant 7 is missing 143 nucleotides in the N-terminal region. **(B)** Map view of sequencing results indicate that no point mutations were found in the coding region (yellow marked arrow) of Ano6/TMEM16F in all cell lines. All sequencing data can be found in SI data 1. Shown are screenshots that were created using the SnapGene^®^ 5.3.1 software (Dotmatics, Boston, MA, USA)

The finding that the Ano6/TMEM16F-KO was not associated with decreased PS externalization rates in all cell lines might be also attributed to the expression of a mutated non-functional Ano6/TMEM16F protein. Thus, Ano6/TMEM16F was fully sequenced all cell lines. The results are summarized in Fig. 7 and show that in all cell lines two transcript variants were expressed. The coding sequence of the full length transcript is 100% identic to the “Homo sapiens anoctamin (ANO6) transcript variant 5 mRNA (accession no. NM_001204803.2)” and codes for a protein with 931 amino acids. The second transcript, which lacks 143 nucleotides in comparison to the full length version is 99.77% identic to the “Homo sapiens anoctamin 6 (ANO6), transcript variant 7, mRNA (accession no. NM_001445831.1) and codes for a protein containing 877 amino acids. Notably, no point mutations in the Ano6/TMEM16F coding regions of both transcripts were observed (Fig. 7; SI data 1). Studies of Scudieri and colleagues showed that the Ano6/TMEM16F V5 transcript variant exhibited a marked PS scramblase activity in HEK293 cells [51]. However, since the authors have not conducted any studies using the V7 transcript variant, it remains unclear what its PS scramblase activity is [51]. Nevertheless, based on these data, we conclude that the Ano6/TMEM16F transcript variant V5 was correctly expressed in all cell lines and should therefore be functional.

### The overexpression of Atp8a1 and its cofactor CDC50a in the cell lines shows some significant effect on Syncytin-1-mediated cancer cell fusion

**Fig. 8:**
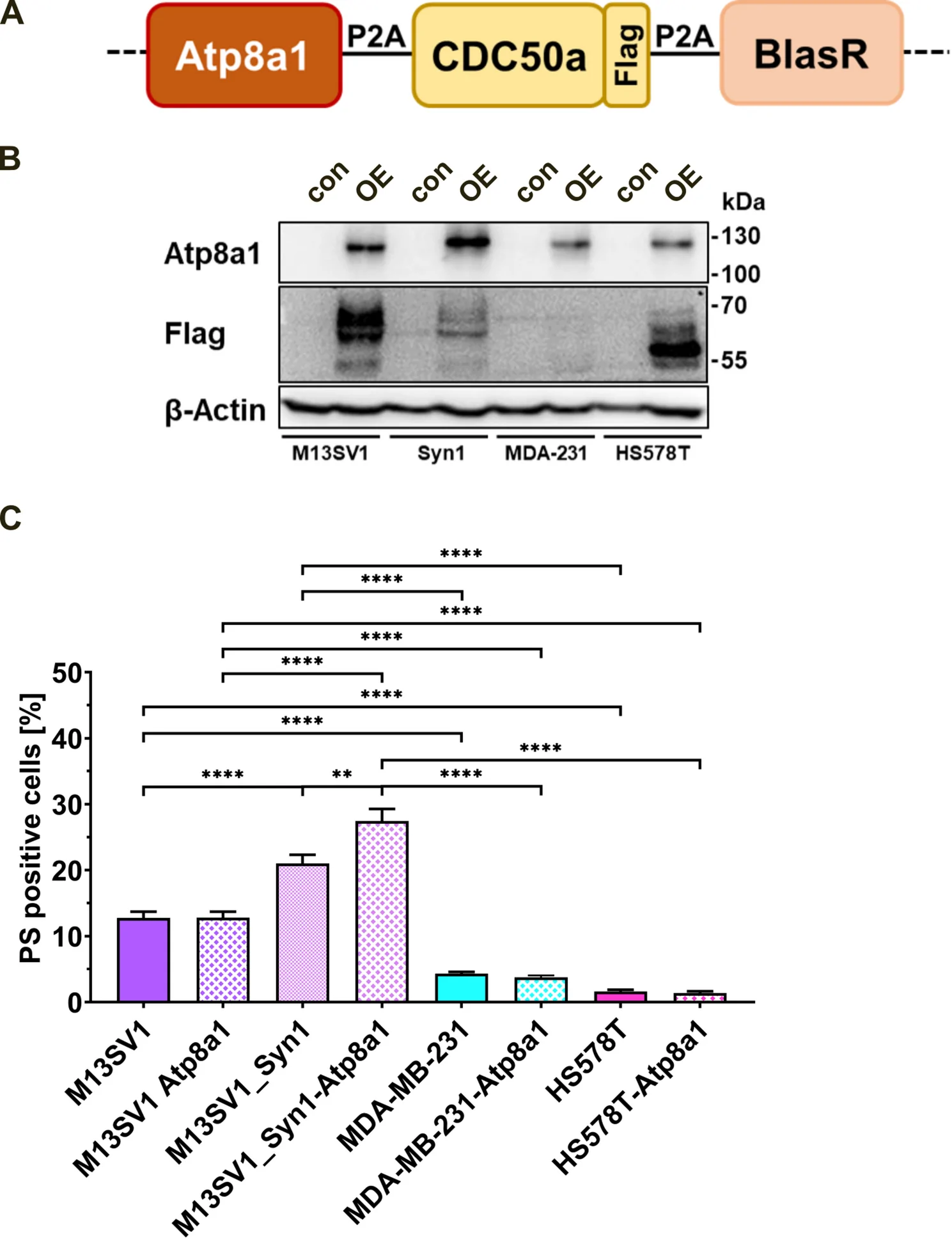
Successful overexpression of Atp8a1 and CDC50a. **(A)** Schematic view of the designed Atp8a1, CDC50a-Flag and a blasticidin resistance (BSD) expression vector. **(B)** Western Blots show the successful overexpression of Atp8a1 in the cells’ membrane fraction and its cofactor CDC50a-Flag in whole cell lysates in M13SV1, M13SV1_Syn-1 (Syn1), MDA-MB-231 (MDA-231) and HS578T cell lines. Shown are representative Western blot data of at least three independent experiments. **(C)** The Atp8a1/CDC50a overexpression had no effect on the cells’ PS externalization levels. Fluorescence microscopy pictures were taken and the amount of all cells was set in ratio to the amount of green fluorescent cells. Shown are the means ± S.E.M. of at least three independent experiments. Statistical analysis was done using an ordinary one-way ANOVA and a Tukey multiple comparisons post-hoc test. *p* ≤ 0.001 = ****. Data with Ionomycin as a positive control (10 µM) are summarized in SI Figure S1. Full-length blots are presented in SI Fig. 4

**Fig. 9:**
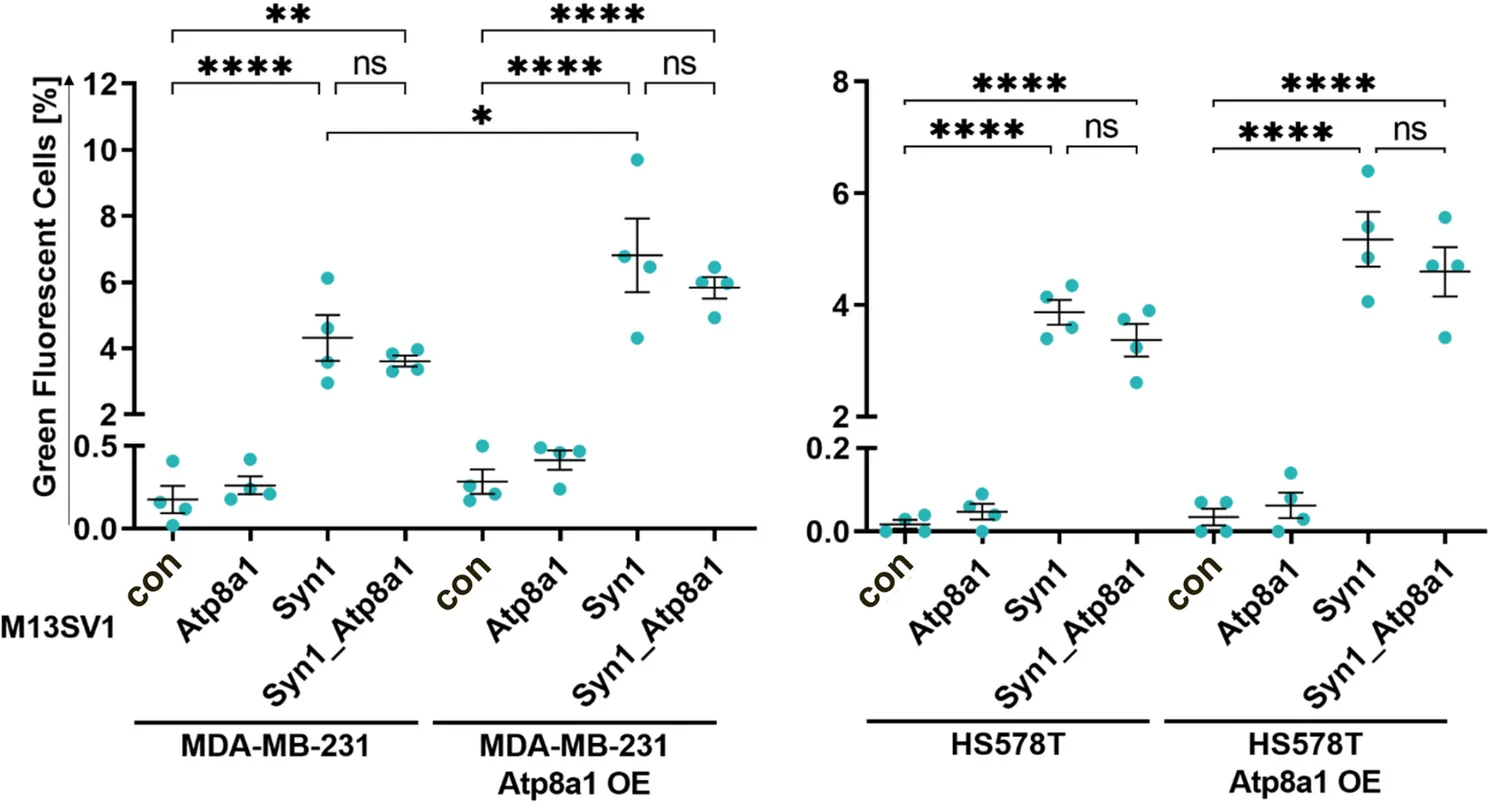
The overexpression of Atp8a1 and CDC50a has no influence of the fusogenicity of cells.** (A)** MDA-MB-231, **(B)** HS578T. The amount of green fluorescent cells was measured by flow cytometry. Shown are the means ± S.E.M. of four independent experiments. Statistical significance was calculated using a one-way ANOVA and Bonferroni’s multiple comparison test. *p* ≤ 0.05 = *; *p* ≤ 0.01 = **; *p* ≤ 0.001 = ***

Since the flippase Atp8a1 may have an influence in the outcome of metastatic breast cancer when dysregulated, it was chosen as a model flippase for the DSP cell fusion assay. Therefore, a vector coding for an Atp8a1-P2A-CDC50a-Flag-P2A-BSD sequence was cloned (Fig. 8A) and transfected into the cell lines used. The overexpression was confirmed by extraction of the membrane fraction for Atp8a1 and whole cell lysates for the detection of the Flag-tagged CDC50a protein for Western Blotting (Fig. 8B). The Western Blots show the overexpression of Atp8a1 (OE) around 120 kDa in the membrane fraction of all transfected cell lines in comparison to the untransfected control cell lines (con). In addition, the expression of Flag and CDC50a, respectively, was detected in the transfected cell lines. Due to different glycosylation patterns, its size varied between 55 and 70 kDa (Fig. 8B). While the protein seems to be highly expressed in M13SV1 and HS578T cells, there is less expression seen in M13SV1_Syn1 and MDA-MB-231 cells. Overexpression of Atp8a1 had no effect on PS externalization in any of the cell lines studied (Fig. 8C). In general, M13SV1 cells and its derivatives (M13SV1-Atp8a1, M13SV1_Syn1 and M13SV1_Syn1-Atp8a1) exhibited significantly higher numbers of PS positive cells as compared to MDA-MB-231 and HS578T cells and its Atp8a1 derivatives (Fig. 8C). We only observed that the amount of PS positive cells was significantly higher in the M13SV1_Syn1-Atp8a1 population as compared to M13SV1_Syn1 cells (M13SV1_Syn1 21.02 ± 1.33% vs. M13SV1_Syn1-Atp8a1 27.46 ± 1.84%; *p* < 0.001; Fig. 8C). These results were rather unexpected, as overexpression of Atp8a1 and CDC50a should actually have resulted in a reduced concentration of PS on the outer surface of the plasma membrane. Next, these genetically modified cell lines were used to set up a heterogenic DSP cell fusion assay. The four M13SV1 cell lines, either combining the control cell line (con), the Atp8a1-CDC50a overexpressing cell line (Atp8a1), the Syncytin-1 overexpressing cell line (Syn1) or the Syncytin-1 and Atp8a1-CDC50a overexpressing cell line (Syn_Atp8a1), with the MDA-MB-231 or HS578T cell line as control cell line or also overexpressing Atp8a1-CDC50a in a ratio of 1:3 for 72 h. The amount of green fluorescent cells was subsequently measured with flow cytometry (Fig. 9). The results of the DSP assay with the breast cancer cell lines as control cells or overexpressing Atp8a1-CDC50a show that cocultures with M13SV1 control cells (con) or Atp8a1-CDC50a overexpressing cells (Atp8a1) yielded in negligible amounts of green fluorescent cells. The coculturing with M13SV1 cells overexpressing Syncytin-1 (Syn1) led to increased amounts of green fluorescent cells, which was slightly decreased, when Atp8a1-CDC50a was overexpressed additionally in M13SV1_Syn1 cells (Fig. 9).

When comparing the values for Syncytin-1 mediated cell fusion between the normal breast cancer cell lines and the breast cancer cell lines overexpressing Atp8a1-CDC50a, there was a slight increase in green fluorescent cells for the cocultures with the Atp8a1-CDC50a overexpression breast cancer cell lines, which again was slightly decreased when M13SV1_Syn1 cells also overexpressed Atp8a1-CDC50a (Fig. 9).

### The KO of Ano6/TMEM16F and the overexpression of Atp8a1 and its cofactor CDC50a in the cell lines show no significant effect on the EMT marker expression, but significantly inhibits colony formation in MDA-MB-231 cells

**Fig. 10:**
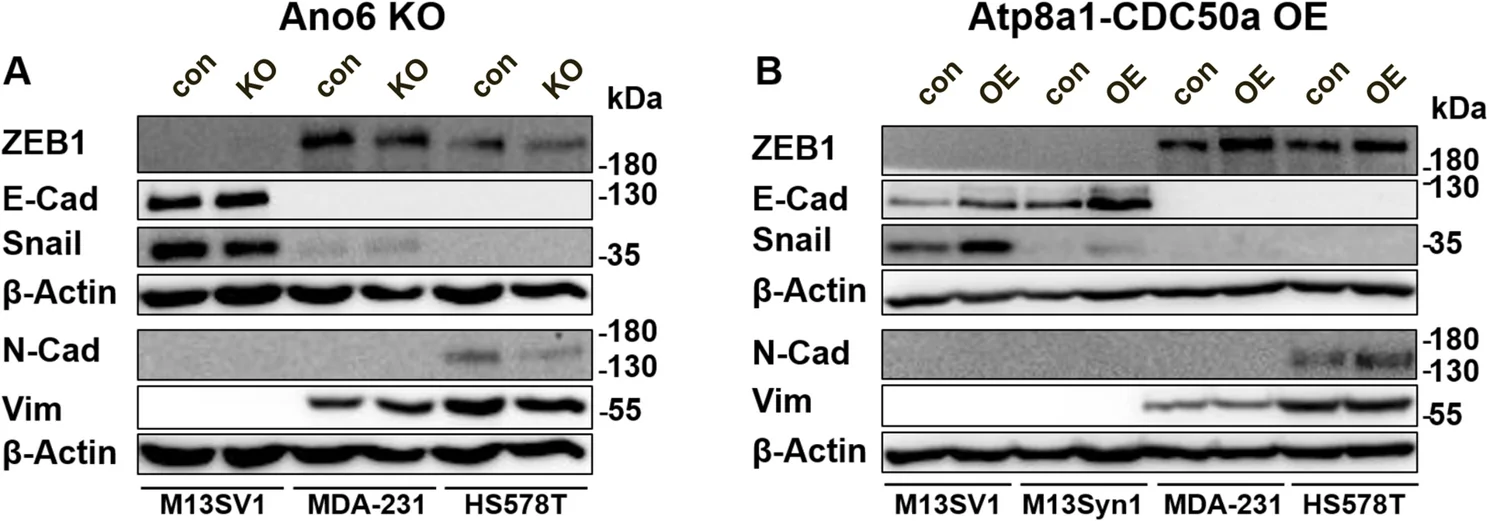
EMT marker expression in control cells and Atp8a1 and CDC50a overexpression cell lines. (**A**) Ano6 knock-out, (**B**) Atp8a1-CDC50a overexpression**.** Shown are representative Western Blot data of at least three independent experiments. The blots were triple or double stained and thus have identical β-actin loading controls. Shown are representative Western blots of at least three independent experiments. Full-length blots are presented in SI Fig. 4

To analyze whether the Ano6/TMEM16F KO or the Atp8a1-CDC50a overexpression, respectively, have an impact on the epithelial-to-mesenchymal-transition (EMT) markers of the cells, Western Blots were performed. The expression levels of Zinc Finger E-box binding homeobox (ZEB1) and Snail as regulators of EMT proteins as well as E-Cadherin (E-Cad, epithelial marker), N-Cadherin (N-Cad, mesenchymal marker) and Vimentin (Vim, mesenchymal marker) were assessed, whereas β-Actin was used as a loading control (Fig. 10A, B). The protein expression profile of the EMT regulators as well as epithelial and mesenchymal markers of the Ano6/TMEM16F KO or Atp8a1-CDC50a overexpression cell lines is rather comparable to control cells (Fig. 10A, B). The Atp8a1-CDC50a overexpression in M13SV1 and M13SV1_Syn1 cells led to a slight increase in Snail and E-Cadherin expression, whereas the Syncytin-1 overexpression itself resulted in an increase in E-Cadherin and a decrease in Snail expression (Fig. 10B). The expression levels of the mesenchymal markers N-Cadherin and Vimentin and the EMT regulator ZEB1 in the breast cancer cell lines were not altered by the Ano6/TMEM16F KO and Atp8a1-CDC50a overexpression (Fig. 10A, B).

### Ano6/TMEM16F KO or inhibition of Ano6/TMEM16F activity inhibition has mostly no significant effect on the cell’s colony formation capacity

**Fig. 11:**
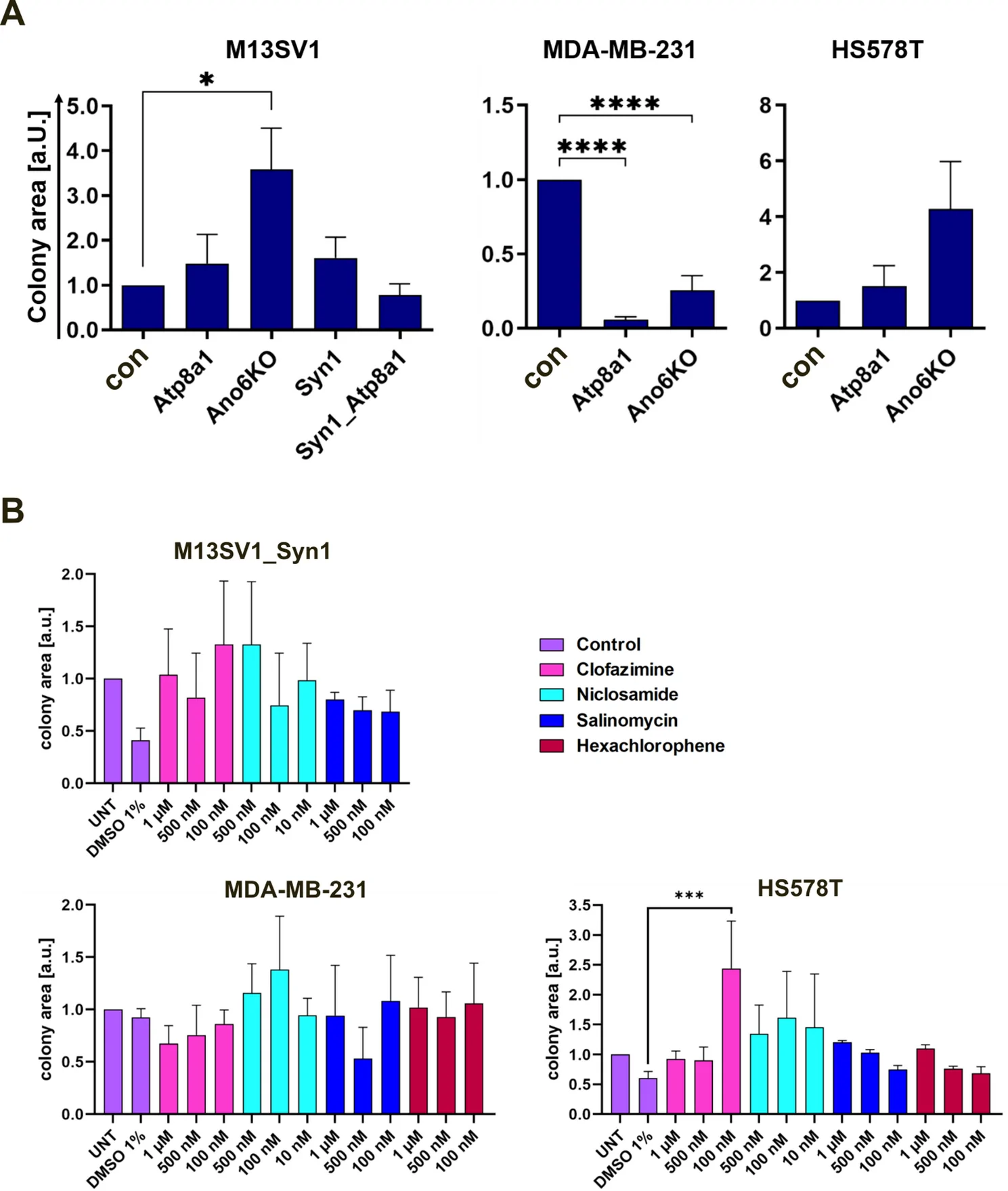
Colony formation assay data**. (A)** In M13SV1 cells no significant effects on the colony formation capacity were seen in the differently modified cell lines compared to wild type cells (WT), except for a significant higher colony forming capacity in Ano6 KO cells. In MDA-MB-231 cells, the Ano6 KO as well as the Atp8a1-CDC50a overexpression lead to a significant decrease in the colony formation capacity and in HS578T cells a trend to a higher colony capacity was seen in Ano6 KO cells. Shown are the means ± S.E.M. of four independent experiments. Statistical significance was calculated using an ordinary one-way ANOVA and Bonferroni’s multiple comparison test. *p* ≤ 0.05 = *; *p* ≤ 0.0001 = ****. **(B)** Relative colony formation of the cells was assessed in the absence or presence of the used substances in different concentrations on M13SV1, MDA-MB-231 and HS578T cells. The untreated control (UNT) was set to one and the other values were put into perspective. Shown are means ± S.E.M. of two (M13SV1 & MDA-MB-231 Salinomycin) or three independent experiments. Statistical analysis was done using an ordinary one-way ANOVA and Dunnett’s multiple comparison test. *p* ≤ 0.001 = ***

To assess whether the overall stemness characteristics of the cell lines were changed by the genetic modifications, the colony formation capacity of the different cell lines was analyzed (Fig. 11A). In M13SV1 cells, an increased colony formation rate was observed only in the Atp8a1-CDC50a, Ano6 KO, and Syn1 variants, but not in the Syn1-Atp8a1 cells (Fig. 11A). Thereby, the M13SV1-Ano6 KO variant exhibited the significantly highest colony formation rate (Fig. 11A). In contrast, the colony formation rate in the MDA-MB-231-Atp8a1-CDC50a and Ano6 KO cells was significantly lower compared to the MDA-MB-231 control cells (Fig. 11A). Similar to the M13SV1 variants, a significantly increased colony formation rate was also observed in the HS578T-Atp8a1-CDC50a and Ano6 KO variants (Fig. 11A). Here as well, the colony formation rate in the HS578T-Ano6 KO cells was markedly, though not significantly, higher compared to that in the HS578T-Atp8a1-CDC50a cells (Fig. 11A). In accordance with the increased colony formation capacity of HS578T-Ano6 KO cells, we also observed a reproducible and significantly higher colony formation capacity for HS578T cells that were treated with 100nM Clofazimine (Fig. 11B). However, no increased colony formation capacity was observed for HS578T cells that were treated with higher Clofazimine concentrations (500 nM and 1 µM). Similarly, a slightly, but not significantly increased colony formation capacity was also observed for HS578T cells that were treated with Niclosamide (Fig. 11B). In contrast to HS578T cells, neither Clofazimine nor Niclosamide had a positive effect on the colony formation capacity of M13SV1_Syn1 and MDA-MB-231 cells (Fig. 11B).

## Discussion

In the present study we investigated the role of the phospholipid PS in the fusion of human breast epithelial cells and human breast cancer. While our data support the impact of PS in cell fusion, the regulation and the translocation to the outer leaflet of the membrane is still less clear.

When adding different media containing TNFα or/and AnxV to the cocultures of M13SV1_Syn1 with MDA-MB-231 cells, different effects on the cell fusion rate were observed (Fig. 1). It is known that cell fusion is increased by inflammatory mediators [52–55] and that TNFα might activate Syncytin-1 expression via the β-Catenin pathway [55]. In this study, Syncytin-1 is already highly expressed in M13SV1_Syn1 cells, which is also associated with a significantly higher frequency of PS positive cells The fact that Syncytin-1-mediated cell fusion was slightly increased in the presence of TNFα may therefore be likely not due to an even greater increase in Syncytin-1 expression, but probably to other TNFα-dependent mechanisms. For example, it was shown that the activity of furin protease, important for cleavage and therefore activation of Syncytin-1, is increased by TNFα [56] which might lead to an increase in cell fusion. Additionally, TNFα is able to induce programmed cell death, leading to irreversible PS exposure on the cell surface [57] and was found to induce Ca^2+^ influx in hepatocellular carcinoma cells [58]. Ca^2+^ is a known trigger for Ano6/TMEM16F that catalyzes the translocation of PS from the inner to the outer leaflet of the plasma membrane, which is mandatory for membrane merger [26, 28]. However, cell fusion is a complex, not yet fully understood process. Thus, it cannot be ruled out that other and even Ano6/TMEM16F independent mechanisms may play a role in the TNFα induced fusion of M13SV1_Syn1 cells. For example, Weiler et al. demonstrated that the TNFα induced fusion of M13SV1 human breast epithelial cells and human MDA-MB-435 cancer cells was attributed to the induction of MMP9 expression [59].

In any case, the finding that the addition of AnxV to the coculturing medium led to a significant decrease in Syncytin-1-mediated cell fusion in the DSP assay is in view with the important role of PS in cell fusion [27]. Since PS exposure on the cells’ surface promotes cell fusion, it is not surprising that AnxV is able to decrease cell fusion by masking PS. However, the detailed role of PS in membrane merger remains unclear so far. Leikina and colleagues proposed that the depletion of PS from the inner leaflet weakens actin cortex-plasma membrane attachments in osteoclasts [60]. As a consequence, the plasma membrane becomes more flexible to facilitate pre-fusion membrane contacts and fusion pore expansion in cell-cell fusion [60]. However, the authors also showed that AnxV rather stabilized the redistribution of PS in the outer leaflet of the plasma membrane [60], indicating that this PS binding protein rather promoted osteoclast fusion. Similarly, Uygur et al. showed that AnxV is required for the fusion of prostate cancer cells [24]. In contrast, Lin et al. demonstrated that AnxV can stabilize the membrane by binding to externalized PS and forming lattice-like structures (50). This slowed down lipid diffusion and greatly increased the order of the membrane, what also restricted cell fusion, for which the membrane needs to be more dynamic and flexible. Thus, the observed reduced fusion capacity in the presence of AnxV may be related to the enhanced stiffness of the cells’ membrane that ultimately impaired cell-cell merger.

An interesting finding was that the frequency of PS positive cells was significantly higher in M13SV1 cells, which particularly applied to its Syncytin-1 overexpressing derivatives M13SV1_Syn1 and M13SV1_Syn1-Atp8a1 cells. Given that both Syncytin-1 and PS promote cell-cell fusion these findings suit well to the cells’ overall increased fusogenicity. However, how Syncytin-1 overexpression is related to or may even cause increased numbers of PS positive M13SV1 cells remains to be elucidated. Given that Ano6/TMEM16F is the most prominent scramblase that is expressed in the cell lines used and due to its pivotal role in cell/membrane merger [26, 28] it may be assumed that the higher amount of PS positive Syncytin-1 overexpressing M13SV1 cells could be attributed to higher Ano6/TMEM16F expression levels. However, Western blot results demonstrated comparable Ano6/TMEM16F protein expression levels in M13SV1 and M13SV1_Syn1 cells. Therefore, increased numbers of PS positive Syncytin-1 overexpressing M13SV1 cells may be either attributed to an enhanced Ano6/TMEM16F activity or to other Ano6/TMEM16F independent mechanisms.

The treatment of the cells with Niclosamide, Clofazimine, Salinomycin and Hexachlorophene, which directly or indirectly impair Ano6/TMEM16F activity [26] neither altered the number of PS positive cells nor impaired the frequency of cell-cell fusion events in this study. Similar results were obtained for Ano6/TMEM16F KO cells. Neither the number of PS positive cells nor the frequency of cell fusion events was altered in comparison to control cells. In fact, the same concentrations of these compounds were used in this study as those used by Braga and colleagues [26]. Therefore, we assumed that Ano6/TMEM16F activity should have been affected, which would have been associated with reduced PS externalization and cell fusion. It cannot be ruled out that higher concentrations of these compounds were necessary to ultimately impair Ano6/TMEM16F activity and even cell fusion. However, this approach was not feasible, as increased cytotoxicity was observed at higher concentrations of the used compounds. Nonetheless, cell fusion studies with M13SV1 cells in coculture with control cells or Ano6/TMEM16F KO cells of MDA-MB-231 or HS578T cell lines showed no or negligible fusion rates with control or ANO6/TMEM16F KO M13SV1 cells. This was quite surprising, since we assumed that less scrambling of PS in the plasma membrane might lead to a reduction of cell fusion. However, PS externalization studies revealed comparable degrees of PS positive cells in control cells and Ano6/TMEM16F variants.

Ano6/TMEM16F sequencing results showed that two transcript variants were expressed in all cell lines that were identic to the Ano6/TMEM16F transcript variants V5 and V7. Notably, no point mutations were identified in the coding regions of both transcripts. It is known that different splice variants of Ano6/TMEM16F, such as V1, V2, V3 and V5, exist, which differ in their N-terminal and C-terminal regions and their subcellular localization [51]. For example, Ano6/TMEM16F V1, V2 and V5 variants differed in the N-terminal region, but were all localized in the plasma membrane [51]. In contrast, the V3 variant differed in the C-terminal region and was mainly localized in the cytoplasm [51]. Interestingly, Scudieri and colleagued showed that the Ano6/TMEM16F transcript variant V5 exhibited a markedly high PS scramblase activity in HEK293 cells [51]. In contrast, the PS scramblase activity of the Ano6/TMEM16F transcript variant V3 was rather low, which was likely attributed to the fact that this variant was mainly localized in the cytoplasm of the cells [51]. In this context, it is also unclear whether the Ano6/TMEM16F transcript variant V7 possesses PS scramblase activity. We were unable to find any further information on this topic in the literature. However, Scudieri et al. also investigated two other Ano6/TMEM16F transcript variants (V1 and V2), which differed from the Ano6/TMEM16F transcript variant 5 (V5) only in the N-terminal region [51]. The transcript variants V1 and V2 also possessed PS scramblase activity comparable to that of the V5 variant. Since the Ano6/TMEM16F transcript variant 7 (V7) also differs from the V5 variant only in the N-terminal region, we therefore assume that the AN06/TMEM16F transcript variant 7 (V7) very likely exhibits PS scramblase activity as well. Therefore, we assume that Ano6/TMEM16F should be functional in all cell lines. However, it still remains unclear why the cell lines nevertheless do not show increased PS externalization rates.

Thus, we assume that the PS externalization in the cells’ was rather independent of Ano6/TMEM16F activity. It is well-known that additional members of the Ano/TMEM16 family exhibit a calcium-dependent PS scramblase activity, such as Ano4/TMEM16D, Ano7/TMEM16G, and Ano10/TMEM16J [61, 62]. In this regard, Ano4/TMEM16D and Ano6/TMEM16F exhibit a comparable high calcium-dependent PS scramblase activity [62]. Whether Ano4/TMEM16D may play a role in PS externalization in the cell lines that have been investigated in this study remains unclear. First of all, Ano4/TMEM16D expression was not analyzed in this study, but it is known that M13SV1 and HS578T cells do express this scramblase (data not shown). Furthermore, Ano4/TMEM16D, like Ano6/TMEM16F, is a calcium-dependent PS scramblase [62]. In this regard, it must be noted that PS externalization levels were only increased slightly in Ionomycin treated MDA-MB-231 cells and its derivates, but not in M13SV1 and HS578T cells and their genetically modified variants. Moreover, the lowest proportion of PS-positive cells was observed in the HS578T cells and its variants. These results therefore suggest that PS externalization was most likely not mediated by calcium-dependent scramblases in the cell lines studied. Otherwise, an increased rate of PS externalization would have been observed in the ionomycin-treated cells. However, it cannot be ruled out completely that insensitivity of M13SV1 cells and HS578T cells and their variants to Ionomycin might be related to different Ca^2+^ thresholds that are needed for Ano6/TMEM16F activation [63]. It is possible that the cells are able to blunt Ca^2+^-peaks, for example with activity of plasma membrane Ca^2+^ ATPases (PMCA) or sarcoplasmic/endoplasmic reticulum calcium ATPases (SERCA pumps), which take them into the ER [63]. Nevertheless, we assume that this possibility is rather unlikely, which also includes the possibility that the cells may have different sensitivities to ionomycin. Colony formation studies revealed that the ability to induce colonies was significantly higher in M13SV1-Ano6 KO and HS578T-Ano6 KO cells, which was partially in agreement with colony formation data of cells that were cultured in the presence of Clofazimine and Niclosamide. It is unclear why Ano6 KO is associated with an increased colony-forming rate of M13SV1 and HS578T cells. Data of Xuan et al. showed that the shRNA-mediated knock-down of Ano6/TMEM16F in U87MG and T98G glioma cells was associated with a significantly reduced colony formation capacity [64]. Thus, the Ano6/TMEM16F knockout should have been rather associated with a reduced colony formation rate, as was the case with the MDA-MB-231 cells. It cannot be ruled out that these are cell line-specific effects associated with the CRISPR/Cas9 knockout, even though several Ano6/TMEM16F knockout M13SV1 and HS578T clones were subsequently pooled. The finding that Clofazimine and Niclosamide may promote the colony formation capacity of cells might be attributed to side-effects. It was found that all substances are polypharmacological, what means they do not exclusively interact with Ano6/TMEM16F, and additionally act as anti-cancer drugs: For example, Niclosamide targets signaling pathways like Wnt/β-Catenin, mTORC or Notch [65–67], and Clofazimine is also able to inhibit Wnt signaling and different receptors [68]. Salinomycin acts as an ionophore, which can transport cations bidirectionally through membranes, inducing the release of Ca^2+^ into the cytosol [69]. Additionally, it has an influence on Wnt/ β-Catenin, mitogen activated protein kinase (MAPK) and Hedgehog signaling [69]. It has also been suggested as a drug for targeting human cancer stem cells [70]. However, it remains to be determined whether the reduced colony-forming rate of the cells in the presence of Salinomycin was attributed to such a mechanism. Hexachlorophene interacts with twelve key human proteins, like MAPK1/3, epidermal growth factor receptor (EGFR) and ERBB2 (HER2), and the SARS-CoV2 spike protein as identified by Alegria-Arcos and colleagues [71]. Another working group found that Hexachlorophene binds to Src Homology Domain-Containing Tyrosine Phosphatase 2 (SHP2), leading to a reversal of the EMT process and inhibition of Ras/MAPK and PI3K/Akt signaling pathways in NSCLC cells [72]. Due to the polypharmacological characteristics of the substances, it is possible that in this study non-monotonic dose responses were observed in HS578T cells. This means that some concentrations used may be too low to induce an effect, while others are too high to target Ano6/TMEM16F, but probably other proteins in the cells. However, it must be noticed that the overall effects of the used compounds on the cells’ colony formation capacity were rather minor to moderate. For this reason, it is unclear whether these are real effects.

Another unexpected finding was that the overexpression of the flippase ATP8A did not lead to a decrease in PS levels in the outer leaflet of the plasma membrane of the cells. While the overexpression of Atp8a1 was clearly stable in all cell lines, the overexpression of CDC50a yielded in different expression levels. The different heights of the bands in the Western Blots are attributed to different glycosylation of CDC50a (it was found to be 55, 65, and 68 kDa big [43]) and differences in CDC50a glycosylation between the cell lines. The rather low expression in MDA-MB-231 cells in comparison to the other cell lines could be due to different cell line specific reasons. Since the exact same plasmid was transfected in all cell lines, its function is out of debate. It is much more due to degradation processes in MDA-MB-231 cells, which may target the mRNA or the mature protein. The finding that Atp8a1-CDC50a and control cells showed comparable numbers of PS positive cells might explain the similar fusion rates of the cells. This, however, would indicate that the flippase Atp8a1 may only play a minor role in the translocation of PS from the outer to the inner leaflet of the membrane. In this regard, additional PS flippases, such as Atp11a or Atp11c, should be investigated [73] and whether they more effectively reduce PS levels in the outer membrane and even cell fusion. To study the opposite effect and investigate cell fusion without the influence of flippases, a KO of CDC50a would also be appropriate, since most flippases are only fully functional and correctly localized with the help of their β-subunit [74, 75]. The loss of CDC50a leads to a constant exposure of PS on the cells’ surface, which does not lead to apoptosis, but functions immunosuppressive [74].

Western blot results revealed increased E-Cadherin and Snail levels in Atp8a1 and CDC50a overexpressing M13SV1 and M13SV1_Syn1 cells, and slightly higher Zeb1 expression levels in Atp8a1 and CDC50a MDA-MB-231 and HS578T breast cancer cells. It is not known why the overexpression of Atp8a1 and CDC50a correlated with a slight increase in the expression of these EMT markers. Li and colleagues demonstrated that the siRNA mediated knock-down of Atp8a1 in non-small cell lung cancer cells was correlated with elevated E-Cadherin and Vimentin expression levels [45], which is contrasting to our results. However, the authors were unable to explain this effect in more detail, so the relationship between Atp8a1 expression and EMT markers remains unclear.

## Conclusions

In the present study we investigated the role of PS in the merger of human breast epithelial cells and human breast cancer cells. Thereby, a particular attention was drawn on the Ano6/TMEM16F scramblase, which play a crucial role in trophoblast fusion and SARS-CoC-2 spike-induced syncytia formation [26, 28]. While the fusion of the cells could be impaired by masking PS with AnxV and which indicates that the merger of the cells depends on externalized PS, neither Ano6/TMEM16F inhibition or its knock-out nor Atp8a1 overexpression resulted in decreased externalized PS levels and a reduced fusion frequency. Thus, it can be concluded that the fusion of human breast epithelial cells and human breast cancer cells is not mediated by Ano6/TMEM16F scramblase activity. In summary, our results underline the complexity of cell fusion. Even though PS has been identified as a the newest member of indispensable cell fusion molecules [27], the biology of PS in cell fusion is very complex and not yet fully understood. In this regard, it must be further considered that the distribution of PS in the plasma membrane is highly dynamic and related to different flip-flop processes [27, 76]. Scramblases catalyzes the transport of PS from the inner to the outer leaflet of the plasma membrane and vice versa, while flippases predominantly catalyzes the transport of PS from the outer to the inner leaflet of the membrane [27, 76]. Thus, additional work is necessary to clarify the role of PS in the fusion of human breast epithelial cells and human breast cancer cells. Ano4/TMEM16D might be good candidate, but it cannot be ruled out that other scrambles might be involved in this process. In this regard, it would also interesting to study why Syncytin-1 overexpressing cells possess a higher proportion of PS positive cells. From a biological point of view the equal presence of Syncytin-1 and externalized PS (both are indispensable for fusion) makes sense to ensure a high cell fusion frequency. In this regard, it would be important to investigate whether this is a general mechanism and, if so, how it is regulated.

## Supplementary Information

Below is the link to the electronic supplementary material.

Supplementary Material 1

Supplementary Material 2

Supplementary Material 3

Supplementary Material 4

## Data Availability

The datasets used and/or analyzed during the current study are available from the corresponding author on reasonable request.

## Abbreviations

Ano6: Anoctamin 6
ANOVA: Analysis of variance
AnxV: Annexin V
ASCT2: Alanine serine cysteine transporter 2
BSA: Bovine serum albumin
Cas9: CRIPSR-associated protein 9
cDNA: complementary DNA
CRISPR: Clustered Regularly Interspaced Short Palindromic Repeats
CS/ICs: Cancer stem/initiating cells
DMSO: Dimethyl sulfoxide
DNA: Desoxyribonucleic acid
DSP: Dual split protein
E-Cad: E-Cadherin
EMT: Epithelial-to-mesenchymal transition
FITC: fluorescein isothiocyanat
GAPDH: Glycerinaldehyd-3-phosphat-Dehydrogenase
GFP: Green fluorescent protein
gRNA: Guide RNA
HERV: Human endogenous retrovirus
KO: Knock-out
PAM: Protospacer adjacent motif
PBS: Phosphate buffered saline
PCR: Polymerase chain reactio
PS: Phosphatidylserine
RNA: Ribonucleic acid
SARS-CoV-2: Severe acute respiratory coronavirus 2
SDS-PAGE: Sodium dodecyl sulfate polyacrylamide gel electrophoresis
Syn1: Syncytin-1
TBS-T: Tris-buffered saline with 1 % (v/v) Tween 20
TMEM: Transmembrane protein
Vim: Vimentin
ZEB1: Zinc finger E-box-binding homeobox 1
