# Supplementary material for: Phosphatidylserine modulation influences syncytia formation in human breast cancer cell lines
Source: BMC Cancer. 2026 Nov 10;26:1412. doi: 10.1186/s12885-026-17341-z (PMC13761059; doi:10.1186/s12885-026-17341-z)

original data – Western Blot Ano6-KO (Figure 5)

chemiluminescence | **blot photo** | **overlay**

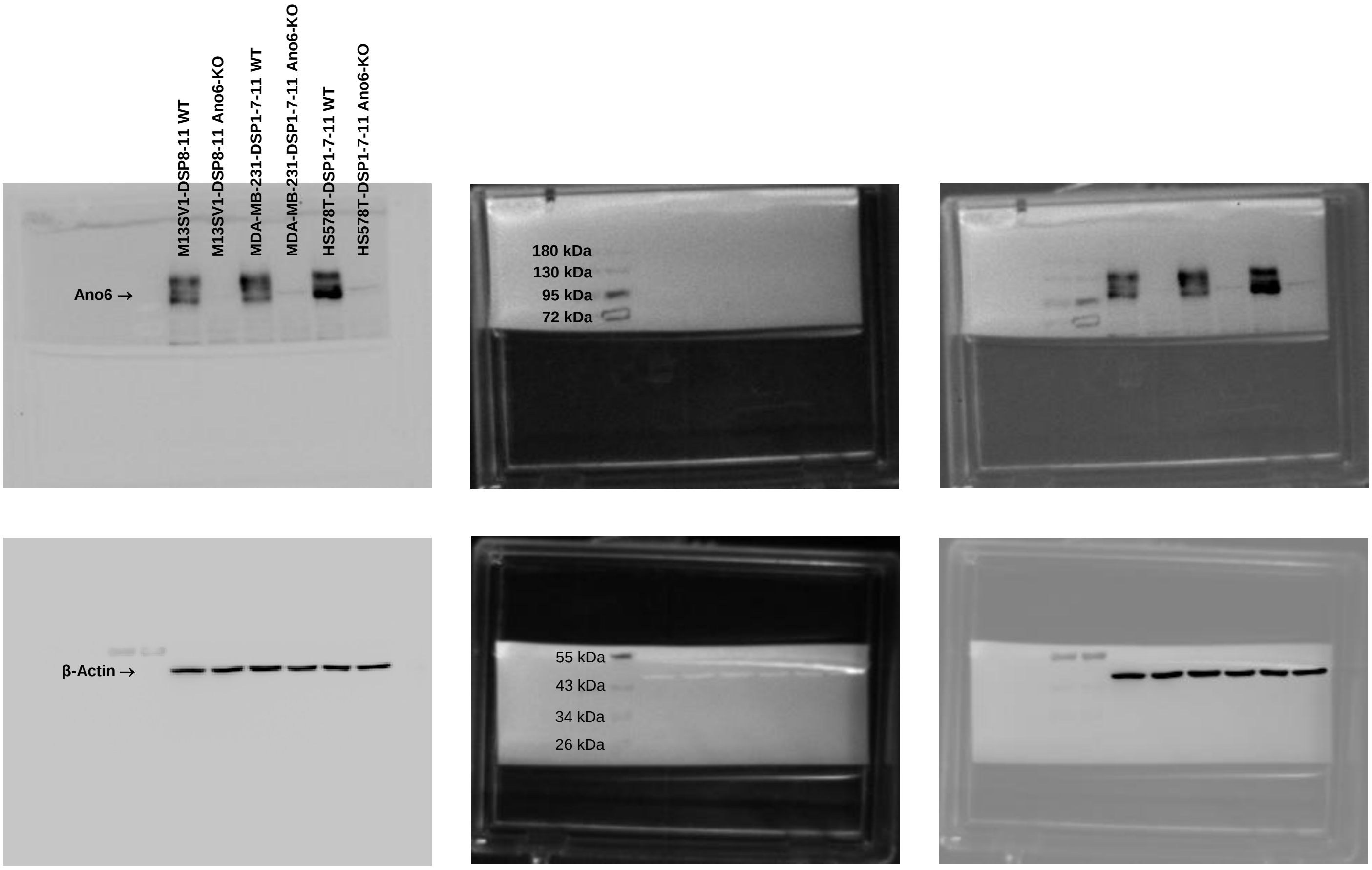

original data – Western Blot Atp8a1 (Figure 8)

chemiluminescence **blot photo** **overlay**

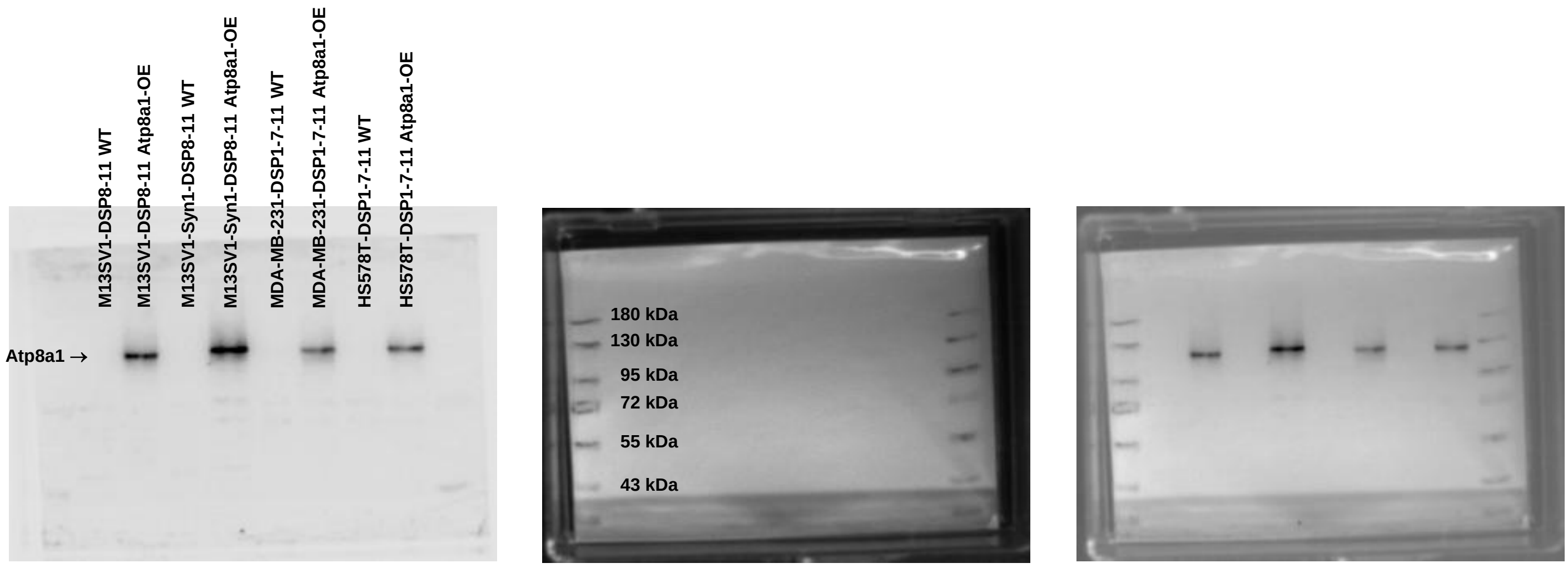

No β-actin loading control was performed because a membrane fraction was used for the detection of Atp8a1.

original data – Western Blot CDC50A/Flag (Figure 8)

chemiluminescence | **blot photo** | **overlay**

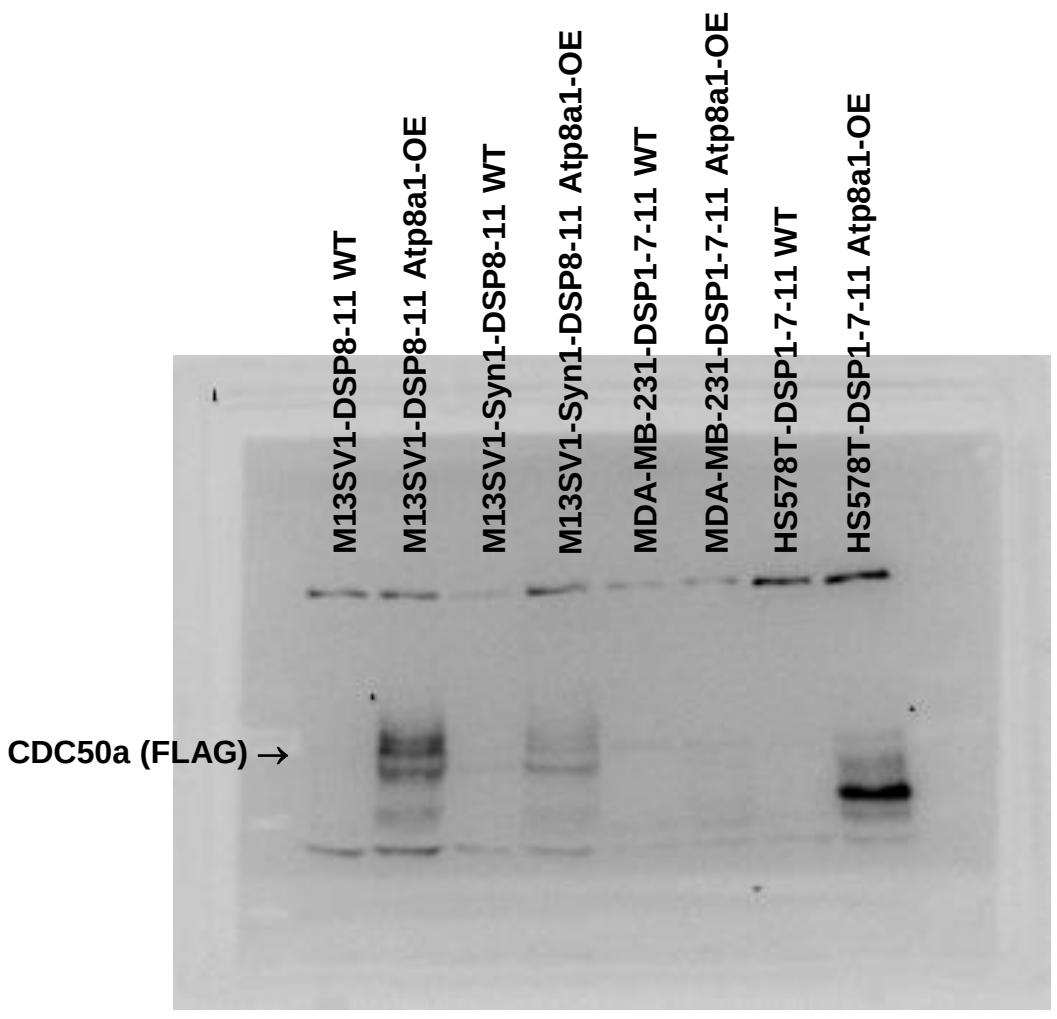

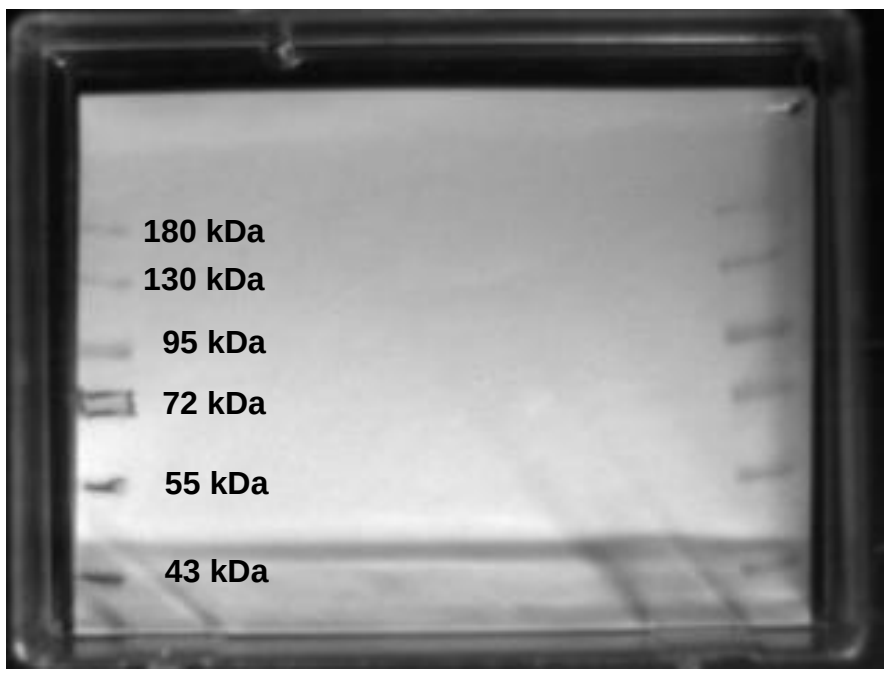

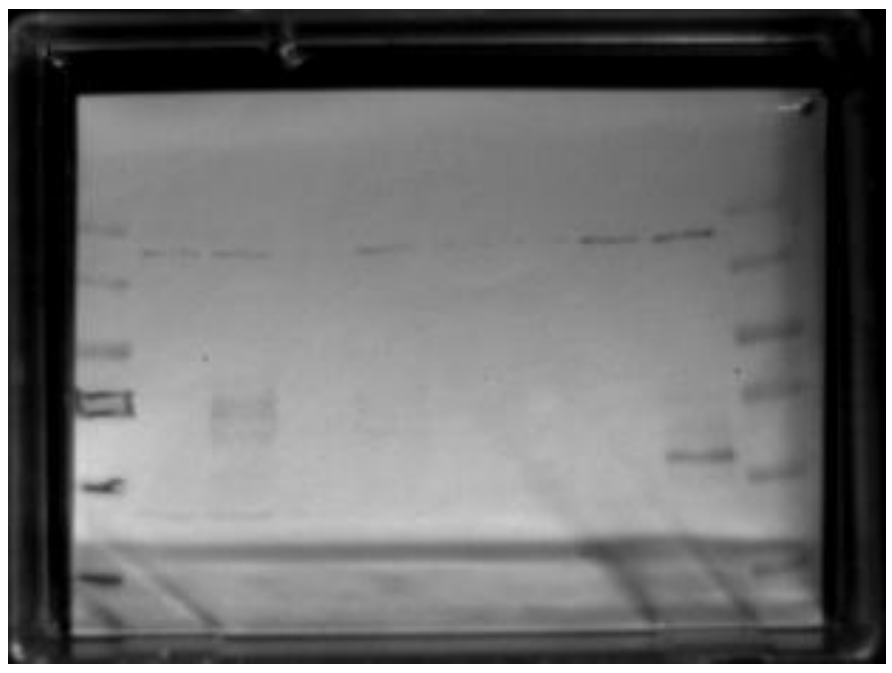

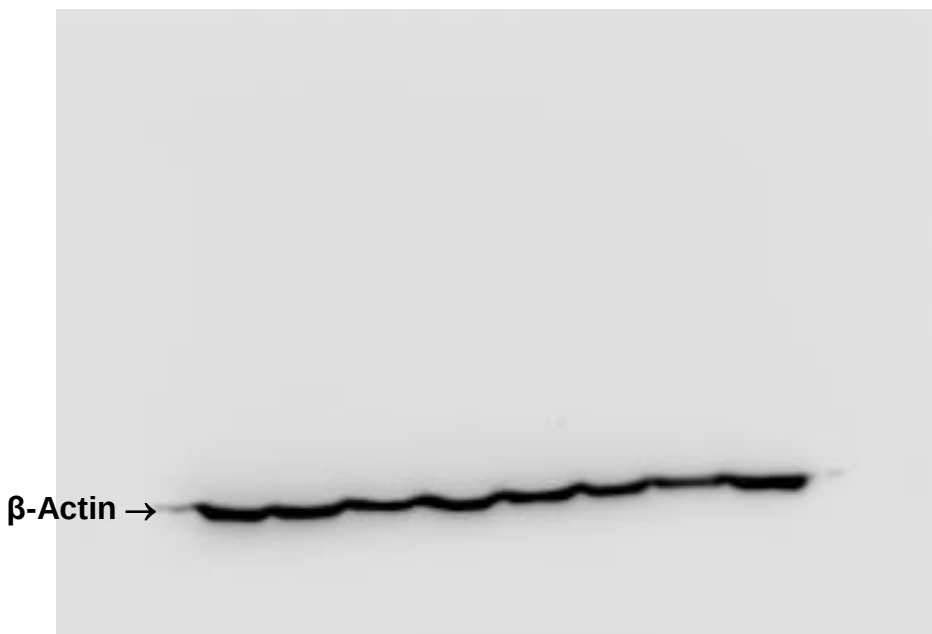

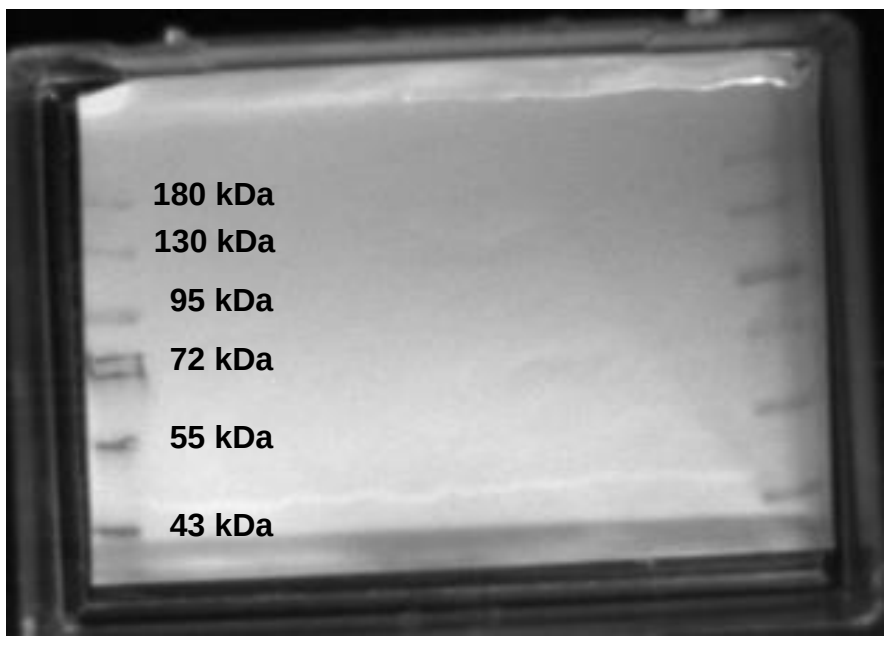

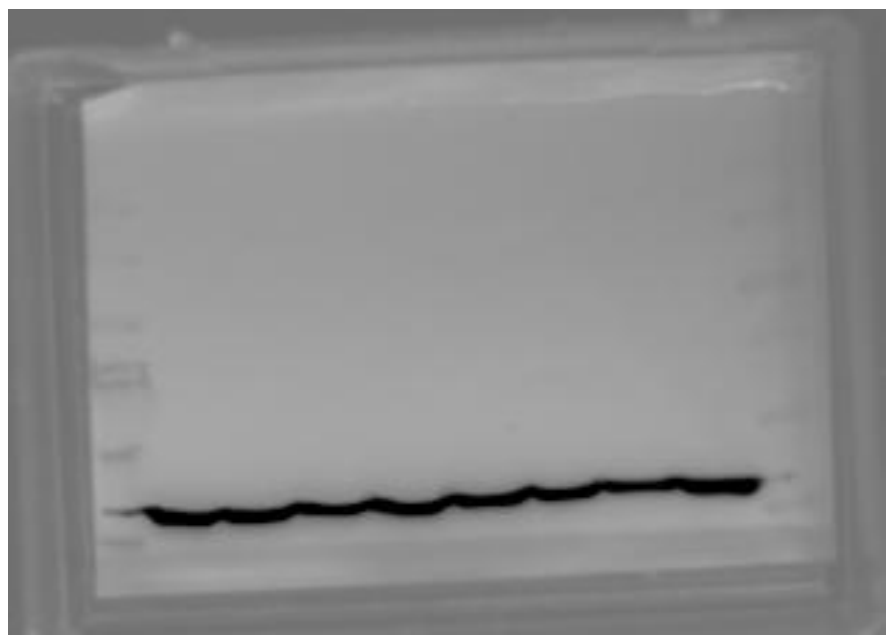

original data – Western Blot Zeb1, E-Cadherin, Snail in Ano6-KO cells (Figure 10)

chemiluminescence | **blot photo** | **overlay**

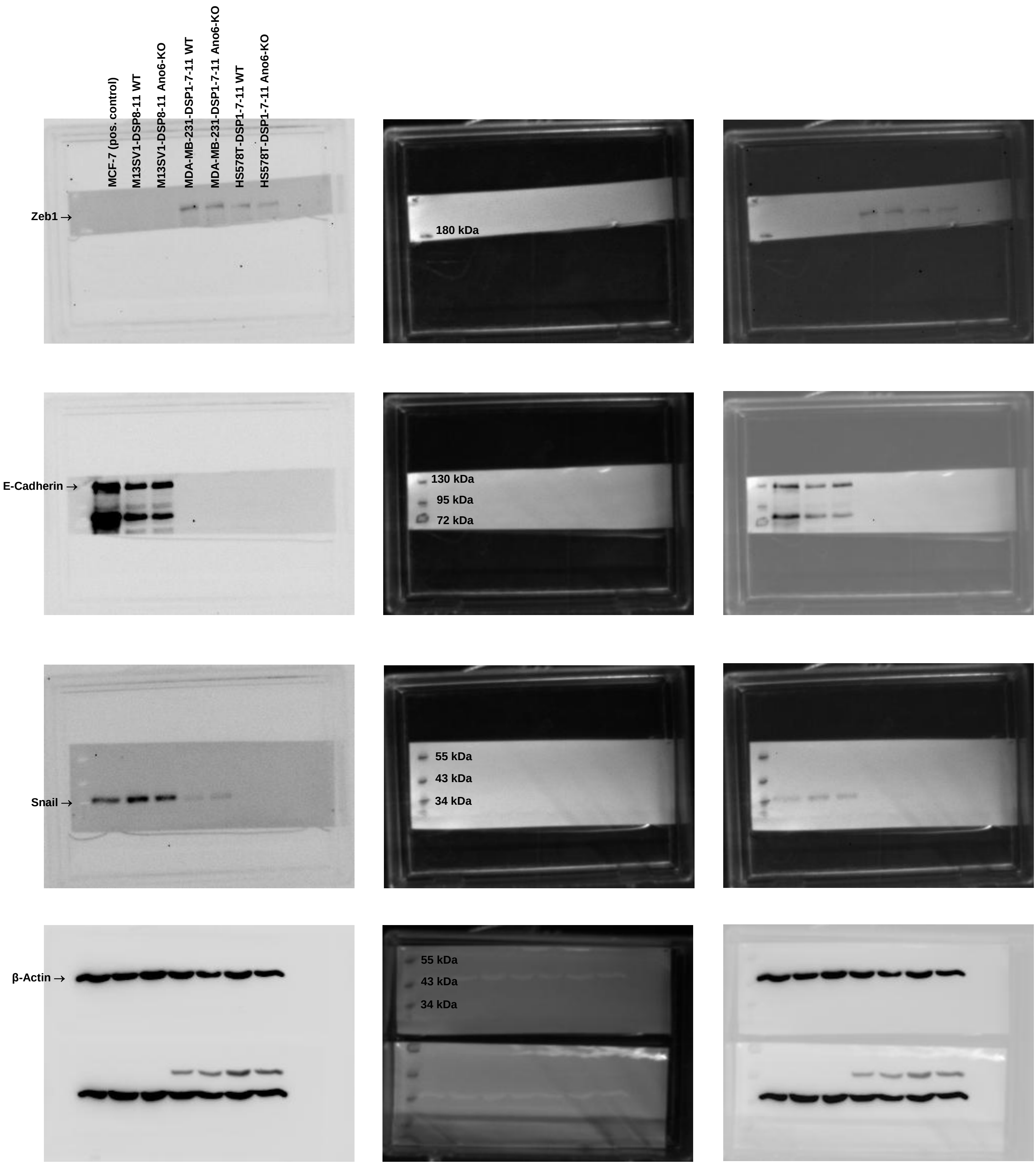

original data – Western Blot Zeb1, E-Cadherin, Snail in Ano6-KO cells (Figure 10)

chemiluminescence | **blot photo** | **overlay**

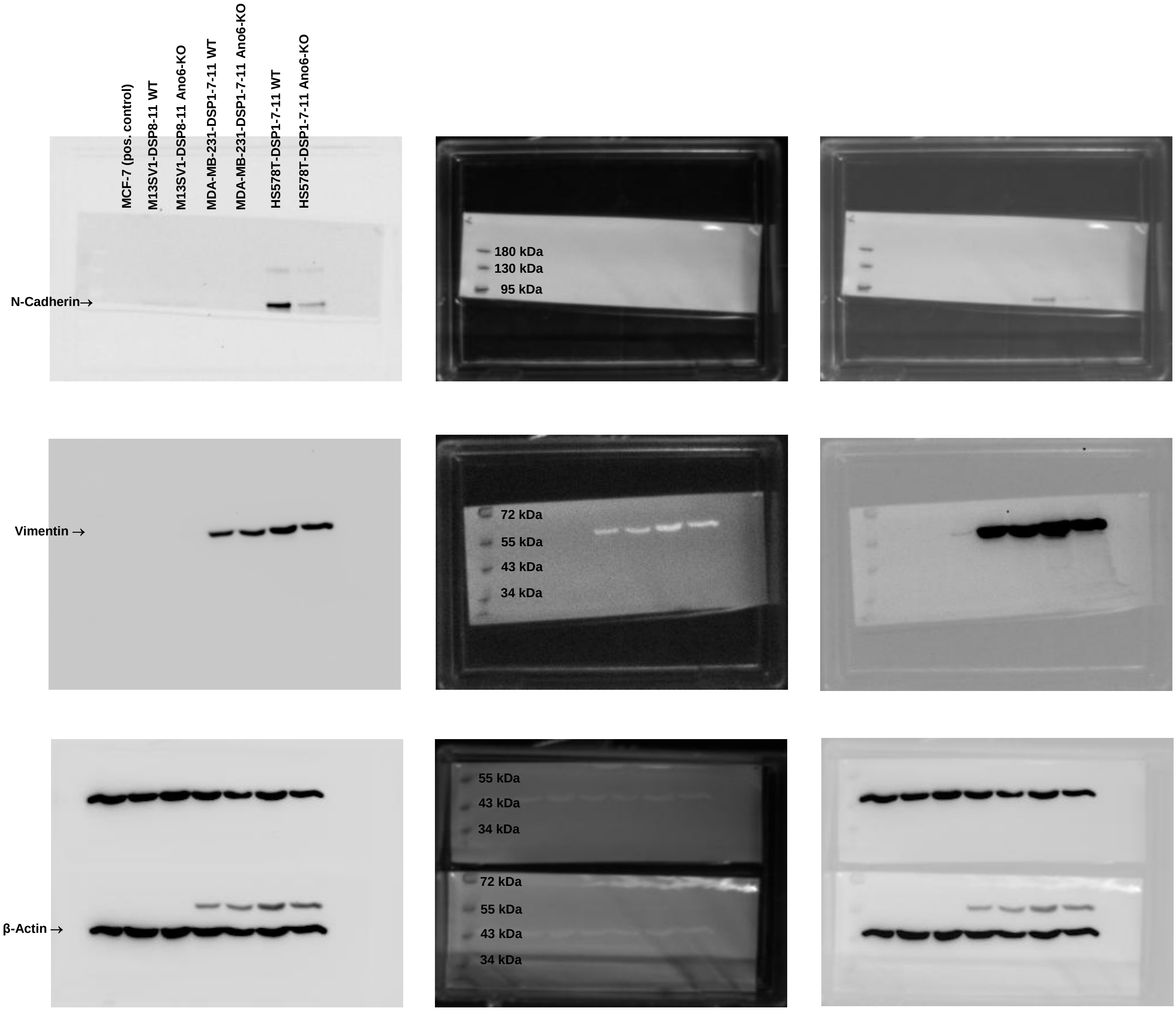

original data – Western Blot Zeb1, E-Cadherin, Snail in Atp8a1-OE cells (Figure 10)

chemiluminescence | blot photo | overlay

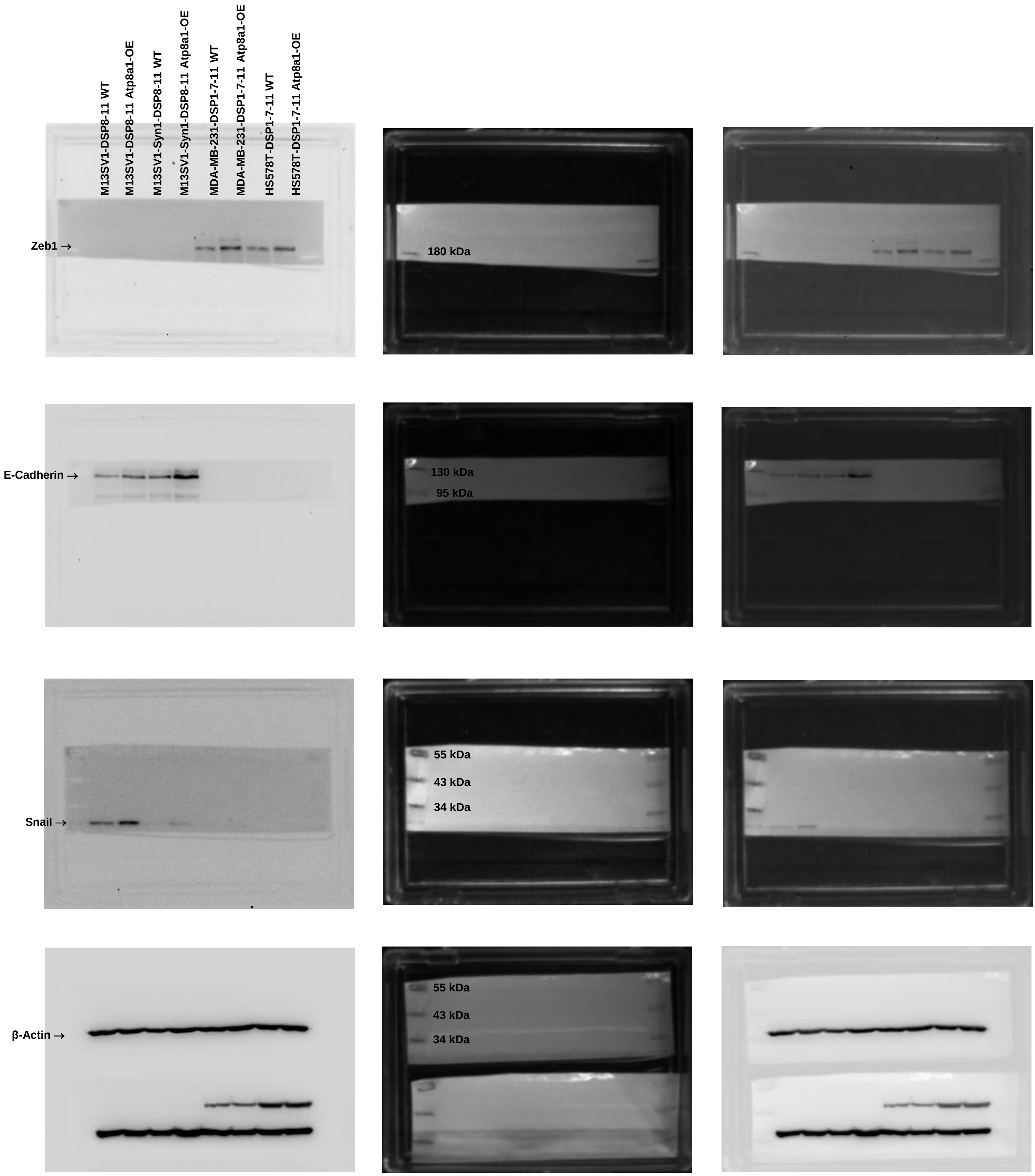

original data – Western Blot Zeb1, E-Cadherin, Snail in Atp8a1-OE cells (Figure 10)

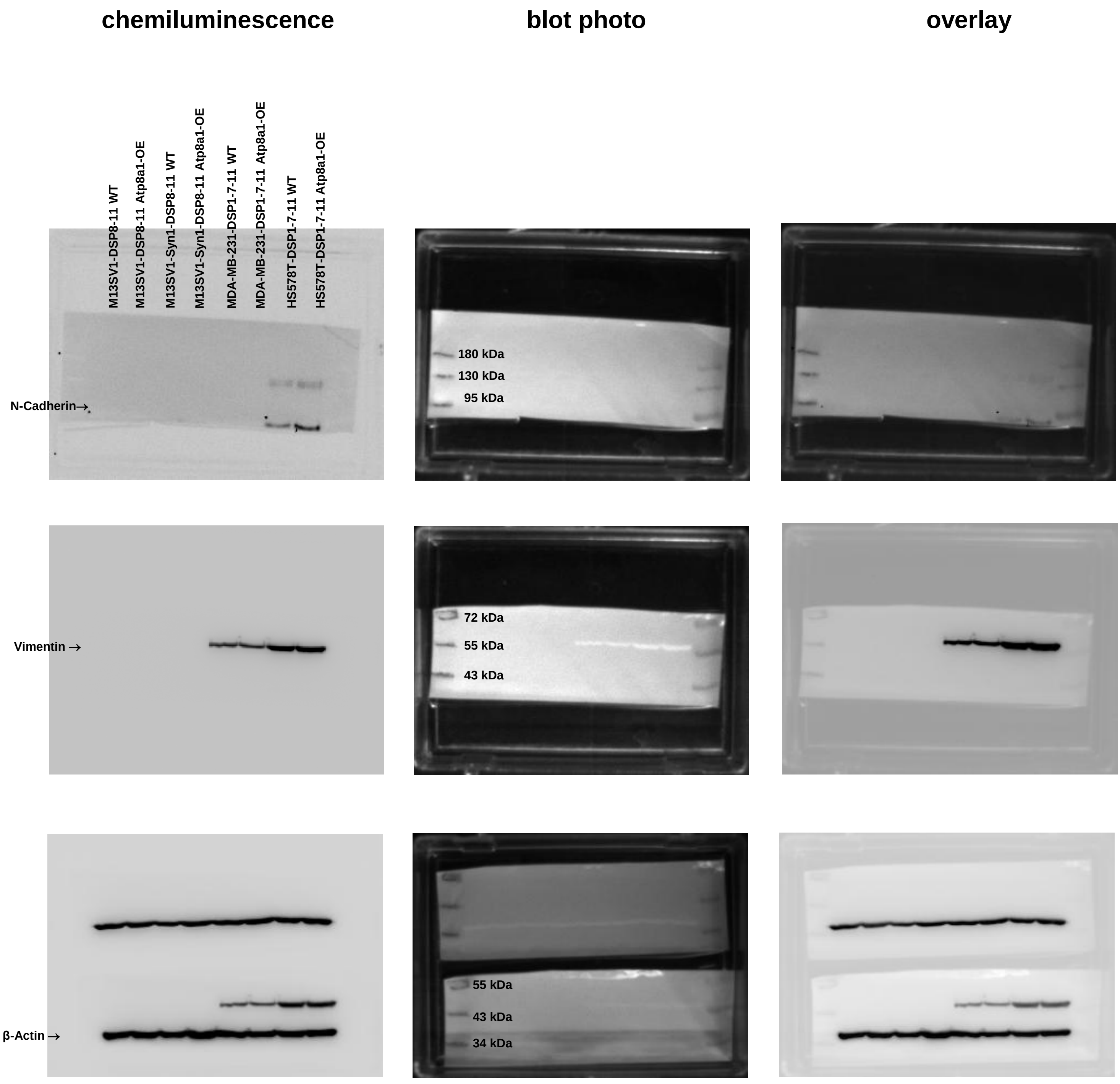

Supplement: Supplementary file 1 [file 12885_2026_17341_MOESM4_ESM.pdf]
