# Supplementary material for: Phosphatidylserine modulation influences syncytia formation in human breast cancer cell lines
Source: BMC Cancer. 2026 Nov 10;26:1412. doi: 10.1186/s12885-026-17341-z (PMC13761059; doi:10.1186/s12885-026-17341-z)
Supplement: Supplementary file 2 [file 12885_2026_17341_MOESM1_ESM.docx]

Supplementary Information

Phosphatidylserine Modulation Influences Syncytia Formation in Human Breast Cancer Cell Lines

**Figure S1. PS externalization results for Figure 5B and Figure 7C including Ionomycin data.** Fluorescence microscopy pictures were taken and the amount of all cells was set in ratio to the amount of green fluorescent cells. Shown are the means ± S.E.M. of at least three independent experiments. Statistical analysis was done using an ordinary one-way ANOVA and a Tukey multiple comparisons post-hoc test. p ≤ 0.01 = ***, p ≤ 0.001 = ****.
